# Cytological and Comparative Proteomic Analyses on Male Sterility in *Brassica napus* L. Induced by the Chemical Hybridization Agent Monosulphuron Ester Sodium

**DOI:** 10.1371/journal.pone.0080191

**Published:** 2013-11-14

**Authors:** Yufeng Cheng, Qian Wang, Zhanjie Li, Jianmin Cui, Shengwu Hu, Huixian Zhao, Mingshun Chen

**Affiliations:** 1 State Key Laboratory of Crop Stress Biology in Arid Areas, Northwest A&F University, Yangling, Shaanxi, P. R. China; 2 College of Life Sciences, Northwest A&F University, Yangling, Shaanxi, P. R. China; 3 College of Agronomy, Northwest A&F University, Yangling, Shaanxi, P. R. China; 4 USDA-ARS and Department of Entomology, Kansas State University, Manhattan, Kansas, United States of America; Moffitt Cancer Center, United States of America

## Abstract

Male sterility induced by a chemical hybridization agent (CHA) is an important tool for utilizing crop heterosis. Monosulphuron ester sodium (MES), a new acetolactate synthase-inhibitor herbicide belonging to the sulphonylurea family, has been developed as an effective CHA to induce male sterility in rapeseed (*Brassica napus* L.). To understand MES-induced male sterility in rapeseed better, comparative cytological and proteomic analyses were conducted in this study. Cytological analysis indicated that defective tapetal cells and abnormal microspores were gradually generated in the developing anthers of MES-treated plants at various development stages, resulting in unviable microspores and male sterility. A total of 141 differentially expressed proteins between the MES-treated and control plants were revealed, and 131 of them were further identified by MALDI-TOF/TOF MS. Most of these proteins decreased in abundance in tissues of MES-treated rapeseed plants, and only a few increased. Notably, some proteins were absent or induced in developing anthers after MES treatment. These proteins were involved in several processes that may be crucial for tapetum and microspore development. Down-regulation of these proteins may disrupt the coordination of developmental and metabolic processes, resulting in defective tapetum and abnormal microspores that lead to male sterility in MES-treated plants. Accordingly, a simple model of CHA-MES-induced male sterility in rapeseed was established. This study is the first cytological and dynamic proteomic investigation on CHA-MES-induced male sterility in rapeseed, and the results provide new insights into the molecular events of male sterility.

## Introduction

Significant heterosis for grain yield and other agronomic traits in rapeseed (*Brassica napus* L.) is well documented [1]. Several approaches to the utilization of heterosis have been proposed, including cytoplasmic male sterility (CMS), genic male sterility (GMS), self-incompatibility (SI) and chemical hybridization agents (CHAs) [2]. CHA-induced male sterility in female parents can provide a rapid, flexible and effective system that can enable the development of hybrids from a large number of parental combinations. CHA-induced male sterility may also circumvent the disadvantages of CMS, GMS and SI approaches, such as environmental instability associated with CMS in maintaining male-sterility and/or male-fertility restoration, high risk of disease susceptibility associated with a narrow cytoplasm genetic background [3], intensive labour to remove half-fertile plants from a GMS female parent [4]. Several dozens of commercial hybrids based on CHA-induced male sterility have been registered according to the bulletins of the Chinese National Crop Variety Approval Committee. Indeed, CHA-induced male sterility is increasingly becoming an important approach for the utilization of heterosis in rapeseed in China.

The establishment of a highly effective, low-pollution CHA approach is critical to the utilization of heterosis. We have found that some structurally diversified herbicides of acetolactate synthase (ALS; EC4.1.3.18; also known as acetohydroxyacid synthase) inhibitors, such as tribenuron-methyl and amidosulphuron, are capable of inducing complete male sterility in rapeseed when applied at a concentration less than 1% of that needed for their herbicidal activities [5], [6]. More recently, we found a new ALS-inhibitor herbicide, monosulphuron ester sodium (MES), that belongs to the sulphonylurea family, designed by Professor Zhengming Li of Nankai University (Tianjin, China), can act as an effective CHA to induce male sterility in *B. napus* [7]. ALS catalyzes the first step of the synthesis of the branched-chain amino acids isoleucine, leucine and valine [8]. ALS is the target of five classes of herbicides, including sulphonylureas, triazolopyrimidines, pyrimidinylthiobenzoates, sulphonylaminocarbonyltriazolinones and imidazolinones [9]. This type of herbicides is widely used for weed control, and the mechanisms by which these herbicides block ALS activity have been extensively studied [10]–[13]. Microarrays have been used to reveal changes in genome-wide gene expression in *Arabidopsis thaliana* after treatment with different herbicides [14]. However, the mechanism of male sterility in high plants induced by these herbicides at low concentrations remains unknown.

In higher plants, the development of the male gametophyte is a well-programmed and elaborate process that plays a crucial role in plant reproduction [15]. Male sporogenous cells in a subset of centrally located anther lobes differentiate and undergo meiosis to produce microspores [16]. The tapetum, located at the innermost sporophytic cell layer that is in direct contact with developing pollens, is a highly active secretory tissue that provides components required for normal pollen development [17]. The timelines for breaking down tapetum are crucial to the viability of pollens [18]–[20].

The developmental process of microgametogenesis is controlled by a coordinated protein network in both somatic and gametophytic cells. To analyze changes in the protein network, two-dimensional gel electrophoresis (2-DE) is widely used to identify differentially expressed proteins during pollen development [21]. Reference protein maps of mature pollens and changes during pollen development have been established in a range of plants, including *Arabidopsis* [22]–[24], rice [25]–[27], tomato [28] and *Lilium longiflorum* [29]. Proteomic analyses have been conducted to analyze changes in protein abundance in plants with male sterility, including cytoplasmic male sterile line in rice [30], Ogura cytoplasmic male sterile line in rapeseed [31], alloplasmic (Tournefortii) cytoplasmic male sterile system in rapeseed [32], photoperiod-sensitive male sterile *7B-1* mutant in tomato [33] and male sterile *ms8* mutant of maize [34]. However, only two studies on CHA-induced male sterility in plants have been reported, and both are on wheat male sterility induced by CHA SQ-1 [35], [36]. The proteomics of CHA-induced male sterility in rapeseed has not yet been reported.

The objective of this study was to uncover the cytological and biochemical mechanisms of MES-induced male sterility in rapeseed. Towards this objective, we investigated the characteristics of MES-induced male sterile rapeseed plants, analyzed morphological changes of microspores and tapetum by comparing differences between normal fertile anthers from control plants and male sterile anthers from MES-treated plants at different developmental stages. We also conducted a comparative proteomic analysis of leaves, little buds, and anthers of control and MES-treated male sterile plants at different developmental stages.

## Materials and Methods

### Ethics Statement

No specific permits were required for the described field studies. No specific permissions were required for these locations/activities. The location is not privately-owned or protected in any way. The field studies did not involve endangered or protected species.

### Chemicals

Monosulphuron ester sodium (MES), a sulfonylurea herbicide used to control broadleave weeds in wheat field, was provided by its designer, Professor Zhengming Li of Nankai University (Tianjin, China), and was firstly exploited as a chemical hybridizing agent of rapeseed by our group. MES can induce complete male sterility in rapeseed when applied at a concentration about 1% of that needed for its herbicidal activity. N,N-dimethylformamide (DMF) and Tween 80 were used as dissolution reagent and surfactant, respectively.

### Plant material and MES treatment

The rapeseed cultivar ‘Zhongshuang No. 9’, developed by the Oil Crops Research Institute of Chinese Academy of Agricultural Sciences (Wuhan, China), was selfed for eight generations before being used for our experiments. Zhongshuang No. 9 was planted in the experimental field of Northwest A & F University, Yangling, Shaanxi, China (108° E, 34°15′ N) during the natural growth season from 2009 to 2010.

The experimental plot contained about 2400 plants grown in 120 rows (2 m long each) at a density of 50 cm space between rows and 10 cm between plants within a row. When the rapeseed plants were at the bolting stage with the longest floral bud ≤2 mm, the plot was divided into two groups each containing 60 rows. Plants in one group were foliar-sprayed with 0.1 µg mL^−1^ MES solution containing 50 ppm DMF and 5 ppm Tween 80 for about 15 ml per plant (about 1% of the concentration that required for it acting as a herbicide in wheat fields to control broadleaf weeds) to induce male sterility during the entire flowering period without affecting the growth and development of other tissues of rapeseed plants based on our preliminary research [7]. Meanwhile, plants in the other group were foliar sprayed with the same amount of solution containing only 50 ppm DMF and 5 ppm Tween 80 as the control.

### Cytological study

When the fertility of the first opened flower of each MES-treated plant was visually detectable for male sterility, the main inflorescences of uniform plants in the MES-treated and control groups were collected into plastic bags and quickly transported to the laboratory on ice. Acetocarmine staining was performed to examine the correlation of the microspore developmental stage with the bud length. Bud samples of the control and MES-treated plants at different microspore developmental stages were treated according to [17]. After treatment, the specimens were sectioned with an Ultramicrotome Leica EM UC7 (Leica Microsystems, Germany). Semi-thin sections (1 µm) were observed and photographed with an Olympus BX51 microscope (Olympus Corporation, Tokyo, Japan) under bright field. Ultrathin sections (70 nm) were observed and photographed with a transmission electron microscope (JEM-1230, JEOl, Tokyo, Japan) on 600 mesh formvar-coated copper grids.

### Plant sample collection for proteomic study

Based on cytological observation results of acetocarmine staining, the collected inflorescence samples of the MES-treated and control groups were classified into three subgroups according to their bud length, namely, small buds <1 mm long (before and during the pollen mother cell (PMC) stage), medium buds 1–3 mm long (from meiosis to the early-uninucleate-microspore stage), and large buds >3 mm long (from the vacuolated-microspore to the mature-pollen stages). In the medium bud and large bud subgroups, anthers were dissected from the buds. Young leaves from the main inflorescences of the MES-treated or control plants were also collected. All samples were prepared on ice, immediately frozen in liquid nitrogen, and then stored at −80°C for later use. Samples harvested from each of the 20 rows of MES-treated and control plants were used as one biological replicate; thus, a total of three independent biological replicates were prepared for each sample in this experiment.

### Protein extraction

Total proteins were extracted according to the TCA–acetone precipitation method as previously described [28], [33]. After drying the collected protein pellets in a vacuum, they were dissolved in lysis buffer (7 M urea, 2 M thiourea, 4% w/v CHAPS, 1% dithiothreitol (DTT), 1% v/v immobilized pH gradient (IPG) buffer (pH 4–7)) by incubating at room temperature for 1 h and centrifuging for 20 min at 20 000× *g*. Protein concentration was determined by the Bradford assay with a series of concentrations of BSA as the standard. The quantified protein samples were stored in aliquots at −80°C.

### 2-DE, gel staining and image analysis

The PROTEAN IEF Cell system (Bio-Rad) and pH 4–7 IPG strips (17 cm, linear; Amersham Biosciences) were used for isoelectric focusing (IEF). About 350 µL of rehydration buffer containing 800 µg of proteins was loaded onto the IPG strip and actively rehydrated for 14 h at 50 V and 20°C. IEF was carried out by applying a voltage of 200 V for 1 h, 500 V for 1 h and 1000 V for 1 h; increased to 10 000 V for over 5 h; and held at 10 000 V until a total of 80 000 V h was obtained. Prior to SDS-PAGE, the strips were equilibrated in 10 ml of reducing equilibration buffer (6 M urea, 1.5 M Tris-HCl (pH 8.8), 20% (v/v) glycerol, 2% (w/v) SDS, a trace of bromphenol blue and 2% (w/v) DTT) for 15 min and in alkylating equilibration buffer containing 2.5% (w/v) iodoacetamide instead of 2% DTT for another 15 min. SDS-PAGE was performed with 11% gels using the PROTEAN II xi Cell system (Bio-Rad). Protein ladders were also loaded on the gel. After SDS-PAGE, the gels were subjected to Coomassie Brilliant Blue staining [37]. Three biological replicates were performed for each sample. Each stained gel was scanned by a UMAX PowerLook 2100XL scanner (UMAX Systems GmbH, Willich, Germany) at 300 dpi. Image analysis was accomplished using PDQuest 8.0.1 software (Bio-Rad Laboratories, Hercules, CA, USA). After automated detection and matching, further manual editing was performed.

Three biological replicates of each sample were used to create replicate groups. Statistical, quantitative and qualitative analysis sets were created between the control and treatment groups. In the statistical sets, Student's *t*-test and 95% significance level were chosen. In the quantitative sets, the upper and lower limits were set to 1.5 and 0.66, respectively. Then, Boolean analysis sets were created between the statistical and quantitative or qualitative sets. The protein spots from the Boolean sets were compared amongst three biological replicates. Only spots displaying reproducible change patterns were considered to be differentially expressed proteins for further MS identification.

### In-gel digestion, MS analysis and database searching

Spots showing significant changes in abundance between the control and treatment were manually selected and excised for protein identification. In-gel digestion of differentially expressed proteins was performed according to [38]. All samples were analyzed using an Ultraflex MALDI-TOF/TOF tandem mass spectrometer (Bruker Daltonics). Mass spectra were acquired by FlexControl (version 3.0, Bruker Daltonics) that recorded in the reflector mode within a mass range of 700–4000 Da.

All results of peptide mass fingerprinting (PMF) were searched in MASCOT version 2.1 (Matrix Science, London, UK) with the following criteria: National Center for Biotechnology Information (NCBI) non-redundant protein database (released data Dec.12, 2012; including 1093002 sequences), species restriction to Viridiplantae (green plants). The other parameters were as follows: enzyme of trypsin; one missed cleavage site, fixed modification of carbamidomethyl (Cys), variable modification of oxidation (Met), peptide tolerance of ±100 ppm, peptide charge of 1+. All peptide masses were assumed to be monoisotopic. The identified proteins had at least five independent peptides matched. The coverage of protein by matched peptides was at least 8%. Only significant hits as defined by MASCOT probability analysis (*P*<0.05), were accepted. The hit with a high score as well as similar relative molecular mass (*M*_r_) and isoelectric point (pI) was selected as the experimental *M*_r_ and pI of the target spot.

## Results

### Characteristics of CHA-MES-induced male sterile rapeseed plants

**Figure 1:**
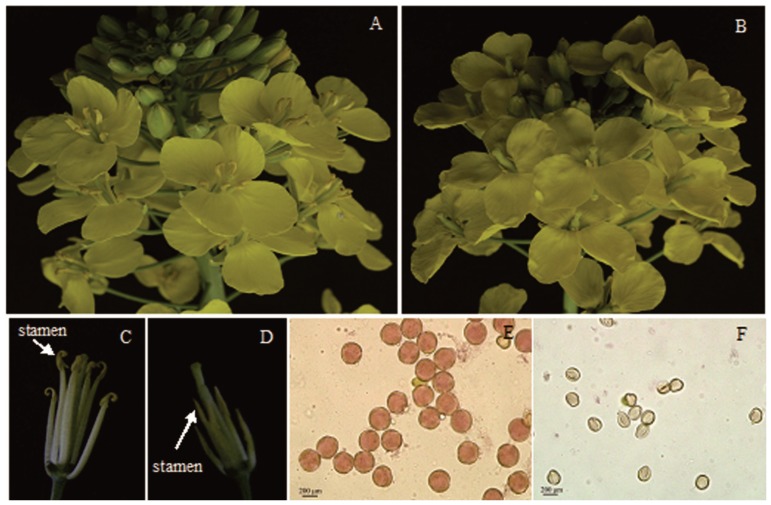
Flowers, stamens and pollens of control plants and MES-treated plants. A, C, E: control plant; B, D, F: MES-treated plants, Viable pollens were stained red by the acetocarmine method (E), but unviable pollens were not stained (F).

Rapeseed plants (with the longest floral bud ≤2 mm) were treated with 0.1 µg mL^−1^ MES at the bolting stage to induce male sterility. The stamens of the MES-treated plants were below the corolla and barely observed from the outside (Figs. 1A and 1B). Closer inspection of the flowers from MES-treated sterile plants indicated that the filament of each stamen did not sufficiently elongate to position its anther at the height of the stigma as that of fertile stamen (Figs. 1C and 1D). The fertile anthers from the control plants produced abundant oval-shaped pollen grains. By contrast, all anthers of MES-treated plants were hollow or shrunken; a few of which produced empty-sac-like pollen grains, but did not dehisce at flowering time. In addition, acetocarmine staining showed that the pollens of sterile anthers from MES-treated plants were unviable (Figs. 1E and 1F).

### Cytological changes in MES-induced male sterile anthers

**Figure 2:**
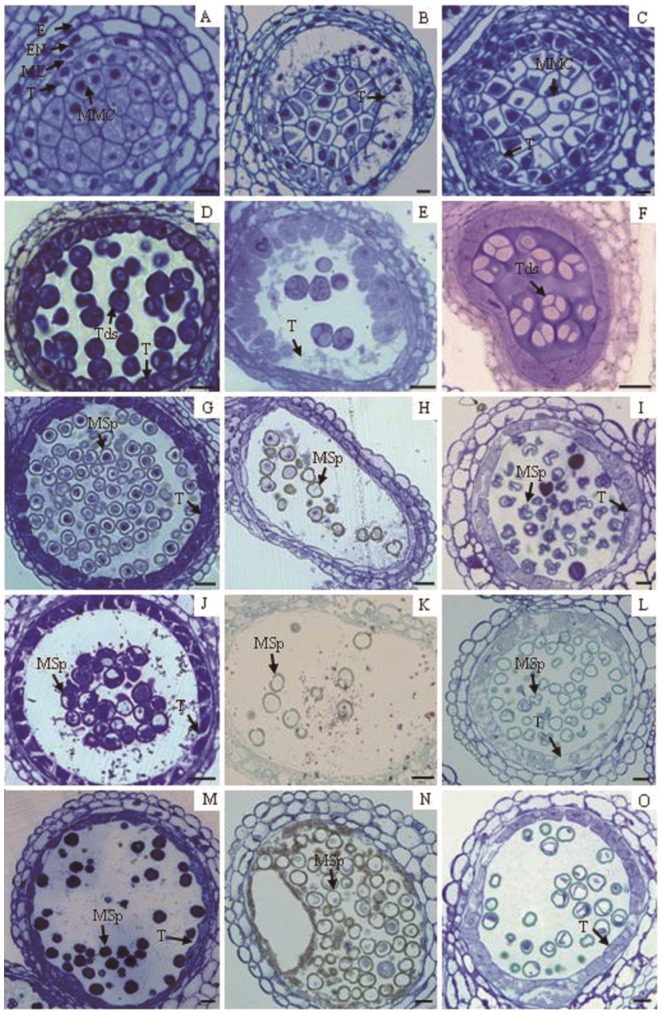
Comparison of the cytological structure of anthers from control plants and from MES-treated plants throughout pollen developmental stages. (A) Normal anthers of control plants at the pollen mother cell (PMC) stage containing microspore mother cells (MMCs) in the centre and four somatic wall layers: epidermis (E), endothecium (EN), middle layer (ML) and tapetum (T). (B) Type-I structure of anthers of MES-treated plants at the PMC stage containing MMCs with condensed bulk in the centre and partial degenerating tapetal cells. (C) Type-II structure of anthers of MES-treated plants at the PMC stage containing MMCs and intact tapetal cells both with condensed bulk in the centre. (D) Normal fertile anther of control plants at the tetrad stage showing tetrads (labelled Tds) and densely stained tapetal cells. (E) Type-I structure of MES-induced male sterile anther at the tetrad stage containing less densely stained tetrads and some loosely arranged tapetal cells. (F) Type-II structure of MES-induced male sterile anther at the tetrad stage containing less densely stained tetrads and tapetal cells. (G) Fertile anther of the control plants at the middle-microspore stage showing normal microspores and tapetum. (H) Type-I structure of MES-induced male sterile anther at the middle-microspore stage showing abnormal microspores (labelled Msp) and disappeared tapetum. (I) Type-II structure of MES-induced male sterile anther at the middle-microspore stage showing abnormal microspores and less stained tapetum. (J) Fertile anther from control plants at the vacuolated-microspore stage showing vacuolated microspores and degenerating tapetum. (K) Type-I sterile anther at the vacuolated-microspore stage showing unviable microspores and disappeared tapetum. (L) Type-II sterile anther at the vacuolated-microspore stage showing unviable microspores and less stained tapetum. (M) Fertile anther of control plants at the mature pollen stage showing mature pollen and degenerating tapetum. (N) Type-I structure of MES-induced male sterile anther at the mature-pollen stage showing unviable pollen and disappeared tapetum. (O) Type-II structure of MES-induced male sterile anther at the mature-pollen stage showing unviable pollen and less stained tapetum.

Comparison of cytological structures between MES-induced male sterile anthers and control anthers throughout different stages was carried out by light microscopy (Fig. 2) and transmission electron microscopy (TEM) (Fig. S1), respectively. At the PMC stage, no apparent cytological difference was observed between the control and MES-induced male sterile anthers in the outermost three somatic layers, *i.e.*, epidermis, endothecium and middle layers (Figs. 2A–2C). However, tapeta defective to different extents were occasionally observed in the anthers of MES-treated plants at the PMC stage, with the two most characterized defective types shown here: type I with more than half of tapetal cells broken down with cell debris left (Fig. 2B); and type II with intact tapetal layers containing the bulk of blue stained stuff condensing together in the centre of cells (Fig. 2C), apparently different from the vacuolated tapetal cells in the control plants (Fig. 2A). The number and shape of microspore mother cells (MMCs) at the centre of type I and type II lobes seemed unchanged compared with those of the control lobes. The cytoplasm of normal MMCs in the control plants were evenly stained, whereas the MMCs of the MES-treated plants with the defective tapetum contained condensed unorganized materials surrounded with a large volume of empty spaces (Figs. 2B and 2C). At the tetrad stage (Figs. 2D–2F), a few less-stained and nucleus-lacking tetrads were sometimes observed in the anthers of the MES-treated plants, whereas plenty of dark-stained tetrads existed in normal anthers of the control plants. Tapeta defective to different extents were also observed in the MES-treated plants at this stage, with two types of the most characterized defective structures shown here: type I with tapetal cells still partially showing interval spaces amongst one another (Fig. 2E), and type II with tapetal cells intact and rectangles resembling the shape in the previous PMC stage (Fig. 2F). At the middle-microspore (Figs. 2G–2I) and vacuolated-microspore (Figs. 2J–2L) stages, abnormal tapetum and deformed microspores were similarly often observed in the anthers of MES-treated plants. Moreover, degeneration of tapetal cells (Figs. 2H and 2K) or delayed dissociation of tapetal cells (Figs. 2I and 2L) in MES-treated plants compared with the normal tapetum in control plants (Figs. 2G and 2J) was observed. At the mature-pollen stage (Figs. 2M–2O), all anthers of MES-treated plants contained abnormal unviable pollen sacs, thus displaying male sterility (Figs. 2N and 2O).

TEM analysis also indicated that although organelles in the abnormal microspores of MES-treated plants at the PMC stage resembled those of the control ones in terms of morphology and number, the plasma membrane did not remain attached to the cell wall (Figs. S1A and S1E). In the abnormal microspores and tapetal cells of the MES-treated plants, various organelles were difficult to observe during all developmental stages (Figs. S1F–S1H, S1M–S1P). In addition, abnormal microspores of MES-treated plants were surrounded by less-developed exines. Finally, structures of sterile pollens from MES-treated plants were apparently distinct from those of mature normal pollen from control plants (Figs. S1Q–S1T).

The above results indicated that defective tapetal cells and abnormal microspores were gradually generated in the developing anthers of MES-treated plants at various anther development stages.

### Changes in the proteomes of MES-treated rapeseed plants

**Figure 3:**
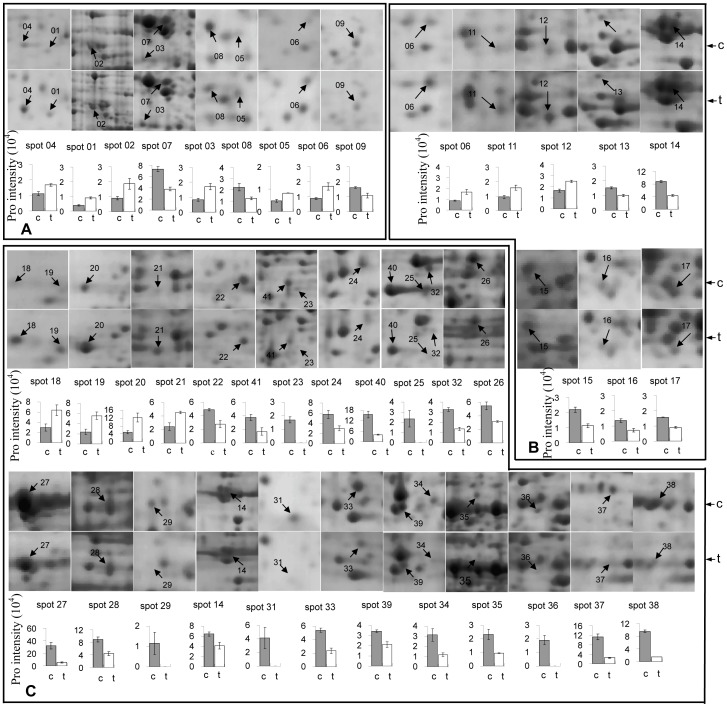
Differentially expressed protein spots in leaves, small buds, anthers from medium buds of control and MES-treated plants. Note: All protein spots are enlarged from Fig. S2 A–F and protein intensity values from Table S1. Protein spot numbers correlate to those in Fig.S2, Table S1 and S2. A, B, C represent differentially expressed proteins in leave, small buds and the anther from middle buds, respectively. c, control, t, MES-treated. Error bars represent standard deviation (n = 3).

To understand the cytological changes in MES-induced male sterile anthers observed above and the mechanism of rapeseed male sterility induced by MES treatment, the changes in proteomes of leaves, small buds, anthers from medium buds and anthers from large buds of the MES-treated and control rapeseed plants were analyzed by 2-DE. Three independent biological replicates were performed in the 2-DE experiment. Representative gel images of protein samples of control and MES-treated plants are shown in Fig. S2. Approximately 1000 spots were detected on each 2-DE gel. Spot-to-spot comparisons and statistical analysis identified a total of 141 spots that exhibited at least 1.5-fold differences in abundance between the control and MES-treated plants (Fig. S2 and Table S1). Specifically, 6, 3, 4 and 23 spots were up-regulated whereas 3, 5, 20 and 77 spots were down-regulated in leaves, small buds, anthers from medium buds and anthers from large buds of MES-treated plants, respectively (Table 1). The 41 differentially expressed spots in leaves, small buds, anthers from medium buds were shown in Fig.3. Most of these differential spots showed quantitative changes, but some showed qualitative changes. For examples, 5 spots (23, 25, 29, 31 and 36) were detected only in anthers from medium buds of the control plants but not in MES-treated plants (Fig.3 and Table S1), 10 spots were detected only in anthers from large buds of MES-treated plants but not in the control plants, and 42 spots were detected only in anthers from large buds of the control plants but not in MES-treated plants (Table 1 and Table S1). The total number of down-regulated spots in the four tissues, especially in anthers from medium buds and anthers from large buds, was much larger than that of up-regulated spots.

**Table 1:** Number of differentially expressed protein spots in various tissues between control and MES-treated plants.

Tissue	Number of up-regulated protein spots a	Number of down-regulated protein spots b	Total number
Leaves	6	3	9
Small buds	3	5	8
Anthers from medium buds	4	15 (5)	24
Anthers from large buds	13 (10)	35 (42)	100

a The number in parentheses represents the number of protein spots detected only in MES-treated plant tissues and not in control ones, which are represented by “+” in the “Fold change” column in Table S2.

b The number in parentheses represents the number of protein spots detected only in control plant tissues and not in MES-treated ones, which are represented by “−” in the “Fold change” column in Table S2.

### Identification and functional classification of differentially expressed proteins

All 141 differentially expressed spots were analyzed by MALDI-TOF/TOF MS. Amongst them, 131 were successfully identified by PMF and are listed in Table S2. All PMF images are shown in Dataset S1. Amongst the 131 identities, 108 (82%) have been functionally annotated in the current database, which represented 81 unique proteins (unipros), whereas the remaining 23 identities were either unknown or hypothetical proteins (Table S2). To infer the possible function of these 23 identities, their sequences were used as queries to search for homologues by BLASTP (NCBI). The corresponding homologues with the highest similarity are listed in Table S3. All hits shared at least 40% sequence similarity, suggesting that they may have similar function with their homologues. These 23 identities represented 23 unipros. Taken together, the 131 identities represented 104 unipros (Table S2 and S3).

**Figure 4:**
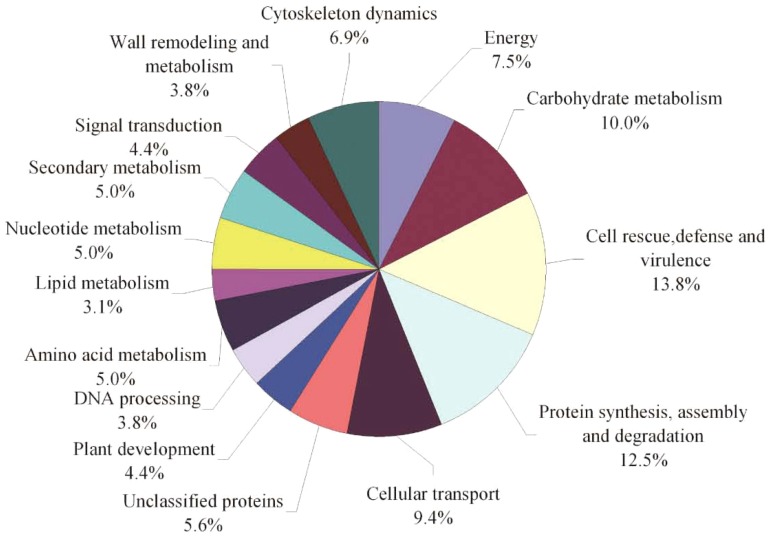
Functional classification of all 104 identified unique proteins. Unclassified proteins include those whose functions have not been described but may be deduced based on sequence homology analysis, as listed in Table S3.

Based on metabolic and functional features, all identified proteins were classified into several functional categories including cell rescue/defence, protein synthesis/assembly/degradation, carbohydrate metabolism, cellular transport, energy, cytoskeleton dynamics, signal transduction, plant development/differentiation, wall remodelling/metabolism, DNA processing as well as amino acid, lipid, nucleotide and secondary metabolism (Fig. 4). Sixty percent of these identified proteins were implicated in the first six functional groups, whereas the first two largest functional groups were proteins involved in cell rescue/defence (13.8%) and protein synthesis/assembly/degradation (12.5%). The other four predominant categories were proteins involved in carbohydrate metabolism (10.0%), cellular transport (9.4%), energy (7.5%) and cytoskeleton dynamics (6.9%). These findings suggested that the above processes were the most affected by MES treatment.

In general, the apparent *M*_r_ predicted by SDS-PAGE has an error of about ±10% compared with the theoretical value (Table S2). However, amongst the 131 identified proteins, a set of 29 identities with known function were found with observed *M*_r_ values much smaller than the theoretical values (Table S2), suggesting that these proteins appeared to be the partially degraded products of their intact proteins. Amongst these identified proteins, seven were suggested to be related to the cytoskeleton dynamic process (a tubulin beta-2 chain, a tubulin beta-9 chain, an actin-1, an actin-3, a dynein heavy chain 8, a dynein-1-alpha heavy chain and a dynein 1b heavy chain), and two identities were related to wall remodelling/metabolism (two UDP-glucose 6-dehydrogenases). A particular case was the tubulin beta-2 chain. All together, four spots (spot 14 in small buds, spots 14 and 19 in anthers from medium buds as well as spot 112 in anthers from large buds) were identified as the same protein, tubulin beta-2, which exhibited different expression patterns in different tissues of MES-treated plants. Although the observed *M*_r_ of spot 19 in anthers from medium buds (17.20 kDa) was much smaller than the theoretical 51.3 kDa, the observed *M*_r_ values of the other three spots (spot 14 in small buds, spot 14 in anthers from medium buds and spot 112 in anthers from large buds) were similar to the theoretical ones. Notably, although spot 19 was up-regulated in anthers from medium buds of MES-treated plants, the other three tubulin beta-2 spots were down-regulated. Apparently, intact tubulin beta-2 was down-regulated in MES-treated plants, and the up-regulated spot 19 represented fragments of tubulin beta-2 that lost their function.

By contrast, nine identities with annotated function were found with observed *M*_r_ values much larger than theoretical values (Table S2), indicating that these proteins may be products of post-translation modified proteins.

## Discussion

ALS inhibitors are among the most important herbicide groups [13]. The biochemical and molecular mechanism by which inhibitors of the sulphonylurea family act as weed herbicides have been investigated [14]. Recently, we found that the new ALS inhibitor MES can be used as an effective CHA to induce male sterility in rapeseed plants at a concentration about 1% of that needed for its herbicidal activity [7]. However, the cytological and biochemical mechanisms of CHA-induced male sterility in plants remain unclear.

Our light microscopy observation combined with electron microscopy analysis indicated that various defective tapeta formed in MES-treated plants throughout the entire anther maturing processes compared with that in control plants. For examples, abnormal tapetum was occasionally observed in anthers of MES-treated plants at the PMC stage, as characterized by more than half of all tapetal cells broken down with cell debris left (Fig. 2B); intact tapetal layers containing the bulk of blue stained stuff condensing together in the centre of cells (Fig. 2C); early degeneration of tapetal cells (Figs. 2H and 2K); or delayed dissociation of tapetal cells (Figs. 2I and 2L) at the middle- and vacuolated-microspore stages compared with normal tapetum in control plants. Apart from defective tapetum, some deformed microspores were also detected in anthers of MES-treated plants at various microspore development stages. Finally, all microspores were unviable at the mature-pollen stage. Abnormalities such as tapetum multiplication, early dissociation and segregate tapetum from mesosphere in other CHA-induced male sterile rapeseed plants have also been previously reported [39]. Based on these observations, rapeseed male sterility induced by MES-treatment could be mainly due to the gradual changes in tapetal cells in MES-treated plants, resulting in various abnormalities throughout the entire tapetum development process. Such abnormalities included early dissociation of tapetal cells, segregated tapetum from mesosphere and delayed degradation. The defective tapetum in MES-treated plants at anther developmental stages cannot supply the components required for normal pollen development, leading to unviable microspores and male sterility in MES-treated plants. These cytological features of male sterility in rapeseed induced by CHA treatment differed from those in CMS [17] and GMS [40], wherein defective tapetum or microspores are generated at a certain developmental stage controlled by specific fertility-related genes.

To date, little is known about the molecular mechanism by which CHA induces male sterility in plant species. To uncover the biochemical and molecular mechanisms of CHA-MES-induced male sterility in rapeseed, leaves, small buds (with anthers before and during the PMC stage), anthers from medium buds (from meiosis to the early-unicleate-microspore stage) and anthers from large buds (from the vacuolated-microspore to the mature-pollen stages) were collected from the MES-treated and control plants to conduct comparative proteomic analysis.

A total of 141 proteins were identified to be differentially expressed between MES-treated and control plants, including 9 in leaves, 8 in small buds, 24 in anthers from medium buds and 100 in anthers from large buds (Table 1, Tables S1 and S2). The nine proteins with altered abundances in leaves of MES-treated plants included the following (Fig.3,Table S2): four up-regulated proteins related to stress responses, *i.e.*, two Glutathione-S-transferases (spot 4, gi|2204102 and spot 6, gi|170177802), an NBS-LRR type resistance protein (spot 5,gi|2792222) and a peroxidase precursor (spot 2, gi|55701025); three proteins involved in protein synthesis, *i.e.*, down-regulated ribosome protein L4 (spot 8, gi|21537296), Ulp1 protease family protein (spot 9, gi|297795029) and up-regulated ribosome protein S4 (spot 3, gi|74273101); and two other proteins, *i.e.*, a down-regulated photosystem II protein (spot 7, gi|49359169) and one up-regulated tubulin fragment (spot 1, gi|54036487). Although the ribosomal protein S4 was up-regulated, the ribosomal protein L4 was down-regulated. The differential regulation of different components of translation machinery suggested a complicated mechanism controlling protein synthesis in response to the stress induced by MES treatment. Up-regulation of proteins involved cell rescue/defence and protein synthesis in response to low-dose MES might be for the purpose to ensure the repair and restoration of growth so that normal growth of vegetable tissues of rapeseed is not significantly affected. This postulation was consistent with the phenotypic change that the leaf colour of rapeseed plants becomes yellow 3 days after MES treatment, but recovers its original colour after 10 days [7].

Comparative proteomic data showed that in small buds with anthers before and during the PMC stage, the following eight proteins were affected in abundance by MES treatment (Fig.3,Tables S2 and S3): five down-regulated proteins, *i.e.*, WD-40 repeat protein MSI4 (spot 15, gi|2599092), tubulin beta-2 (spot 14, gi|297819272 in small buds), thioredoxin M-type (spot 13, gi|11135407), N-glyceraldehyde-2-phosphotransferase-like (spot 16, gi|8885622) and a retrotransposon protein (spot 17, gi|77554545); and three up-regulated proteins, *i.e.*, glutathione S-transferase (spot 6, gi|170177802), ethylene overproducer 1 (spot 11, gi|240255605) and an integrase/recombinase (spot 12, gi|51535085). The WD-40 repeat protein family comprises numerous members with important roles in various cellular functions such as cell growth, proliferation, apoptosis and intracellular signal transduction [41]. WD40 expression is also reportedly related to H_2_O_2_ stress [41]. Reduced activity of an *Arabidopsis* WD40 repeat protein interacting with the tubulin complex can result in largely defective male gametophytes [42]. In addition, decreased WD40-MSI4 expression can delay flowering time, which was also observed in our previous experiment [7]. This phenomenon can be attributed to the interaction of MSI4 with CUL4-DDB1 involved in ubiquitin and PRC2-like complex involved in epigenetics to control the epigenetic regulation of flowering time in *Arabidopsis* [43], [44]. Although only a few proteins changed in abundance in small buds of MES treated plants, obvious defective tapetal cells and abnormal PMCs were observed (Figs. 2B and 2C). The defective tapetal cells and abnormal PMCs in small buds of MES-treated plants probably resulted from the down-regulation of a few key proteins required for microspore development, such as WD-40 repeat protein MSI4 and tubulin.

Interestingly, in anthers from medium buds of MES-treated plants, some key proteins required for microspore and tapetum development were also found to decrease in abundance or be absent compared with control plants. Consequently, defective tapetum and deformed microspores formed at the middle- to vacuolated-microspore stages of anthers (Figs. 2H, 2I, 2K and 2L) A total of 24 proteins were found to be differentially expressed in anthers from medium buds of MES-treated plants compared with the control plants (Fig. 3, Tables 1 and S2). Notably, 20 of the 24 proteins decreased in abundance. Amongst them, five were absent in MES-treated plants (Fig.3,Tables 1 and S2), including plant development/differentiation-related protein phytochrome B-1 (spot 23, gi|312231793), cytoskeleton dynamics-related protein dynein1-alpha heavy chain (spot 31, gi|30580468), cellular transport related protein Mu1-Adaptin (spot 29, gi|159476424), ribulose-1,5-bisphophate carboxylase/oxygenase large subunit (spot 25, gi|125972284) and spermidine synthase (spot 36, gi|297792679) (Tables S2 and S3). Further analysis indicated that all 20 down-regulated proteins can be classified into two groups. The first group consisted of regulatory proteins such as proteins involved in histone methyltransferase and those related to methyltransferase (spots 37, gi|145355325, 33, gi|30688506, 34, gi|30688506 and 35, gi|30688506), RNA processing (spot 38, gi|18415850) and translation (spot 32, gi|132270). The second group consisted of proteins that mainly functioned in plant development/differentiation, cell wall remodelling, cytoskeleton dynamics, cellular transport and metabolism of lipid, carbohydrate as well as energy. Amongst these functional proteins, reversibly glycosylated polypeptide (RGP; spot 22, gi|15237362), Hmg-Coa synthase enzyme (spot 26, gi|112490556) and chalcone synthase (spot 28, gi|294845743) decreased in abundance by 1.7-fold to 2.0-fold in anthers from medium buds of MES-treated plants. Previous studies have shown that the individual disruption of Mu1-adaptin in clathrin vesicle coat in intracellular transport, proteins for tapetum-specific metabolism (Hmg-Coa synthase enzyme in isoprenoid synthesis and chalcone synthase in flavonoid synthesis) and RGPs in polysaccharide biosynthesis in cell walls can result in male sterility in *Arabidopsis* [45]–[48]. All these proteins important for microspore and tapetum development significantly decreased or were absent in anthers from medium buds of MES-treated rapeseed in the present study.

Furthermore, 100 proteins were found to be differentially expressed in anthers from large buds (from the vacuolated-microspore to the mature-pollen stages) of MES-treated plants, with 77 of them down-regulated and 23 up-regulated (Tables 1 and S2). Amongst the 77 down-regulated proteins, 42 were absent in MES-treated plants; amongst the 23 up-regulated proteins, 10 were newly induced in MES-treated plants. Further analysis showed that most of the 23 up-regulated proteins in anthers from large buds of MES-treated plants were related to the degradation of carbohydrates, lipids and DNA. By contrast, numerous proteins involved in cell rescue/defence, wall remodelling/metabolism, cytoskeleton dynamics, cellular transport, and biosynthesis of protein, carbohydrates, lipid and DNA were down-regulated. This down-regulation was accompanied by low energy production and decreased requirement for redox homeostasis maintenance in anthers from large buds of MES-treated plants. As expected, the above changes were detected in anthers from large buds (anthers from the vacuolated-microspore to the mature-pollen stages) of MES-treated plants because normal mature pollen grains store a number of substances (*e.g.*, polysaccharides, proteins, lipids and hormones) that place a high demand on energy and carbon reserves for successful germination and tube growth.

**Figure 5:**
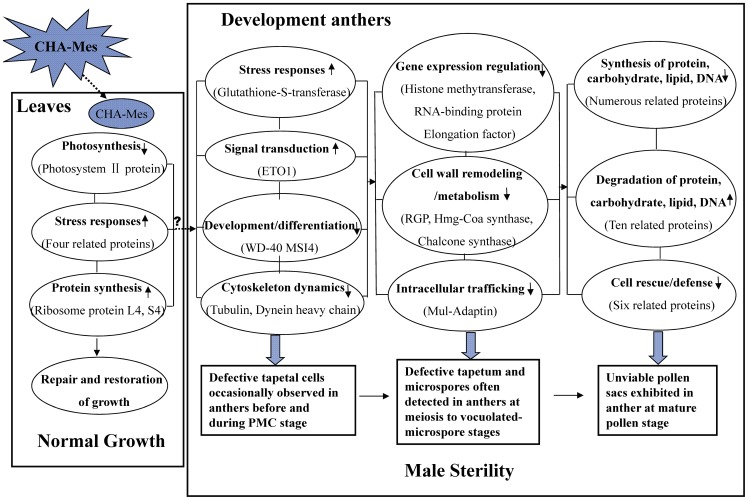
A simple model of CHA-MES-induced male sterility in rapeseed. Only some important differentially expressed proteins in leaves and developing anthers are listed in parentheses (see text for details). ↑ in ovals means up-regulation; ↓ in ovals means down-regulation. Three piles of oval in the ‘Developing anthers’ box exhibited changes in biological processes in small buds with anthers before and during the pollen mother cell stage, anthers from medium buds from meiosis to the vacuolated-microspore stage, and anthers from large buds from the vacuolated-microspore to the mature-pollen stages, respectively. Three rectangles in the ‘Developing anthers’ box showed the cytological features observed in the three corresponding anther developmental stages. ‘?’ in the ‘Leaves’ box represents unclear signals.

Based on these cytological and proteomic analysis results, a simple model of CHA-MES-induced male sterility in rapeseed was established (Fig. 5). Although MES was absorbed by rapeseed leaves before flowering, leaf tissues relatively grew normally by stress responses, repair and restoration of growth. However, newly developing anthers can perceive small changes in homeostasis transported from leaves by some unknown signals, modifying their proteome to adapt to this condition. Initially, a few key proteins required for plant development/differentiation and cytoskeleton dynamics were down-regulated in small buds, resulting in defective tapetal cells occasionally observed in anthers before and during the PMC stage. Then, some proteins important for gene expression regulation, cell wall remodelling/metabolism and intracellular trafficking were down-regulated in anthers from medium buds, leading to defective tapetum and deformed microspores often detected in anthers from meiosis to the vacuolated-microspore stage. Finally, some proteins involved in the degradation of protein, carbohydrate, lipid and DNA were up-regulated in anthers from large buds, whereas numerous proteins related to the synthesis of these macromolecules were down-regulated. Consequently, abnormal unviable pollen sacs formed and the MES-treated rapeseed plants exhibited male sterility.

Differential proteomic analyses on floret intact chloroplast and pollen grain proteins in a wheat male sterile line induced by CHA-SQ-1 have identified six and seven differentially expressed proteins, respectively [35], [36]. Amongst these proteins, only one was found in our data (ascorbate peroxidase) probably because of the different tissues and CHAs used in the present and previous experiments. Firstly, expressed proteins from floret intact chloroplasts and pollen grains were not likely to overlap much with those from leaves and developing anthers. Secondly, CHA-MES and CHA-SQ-1 are two totally different chemicals that act in different ways. In addition, two proteomic analyses in cytoplasmic male sterile anthers in rapeseed have been reported: one in 2 mm Ogura CMS anthers with detailed protein profiles and changed expression [31], and another in alloplasmic (Tournefortii) CMS anthers without protein identification [32]. Comparison of the present proteomic data on anthers from medium buds (1–3 mm long) of MES treated plants with previous proteomic data on Ogura CMS anthers (2 mm) provided useful information. Firstly, both chalcone synthase for flavonoid synthesis and tubulin for cytoskeleton composition have been identified in both investigations. Secondly, various proteins in both cell wall synthesis and stress response have been identified. Thirdly and most interestingly, the expression of numerous proteins involved in the metabolism of carbonhydrate, protein and energy changed in Ogura CMS anthers but not in the present anthers from medium buds of the MES treated plants. This suggests that the biochemical and molecular mechanism of CHA-MES induced male sterility in rapeseed is different from that of Ogura CMS in *B. napus* controlled by the mitochondrial gene *orf138*.

Unexpectedly, no abundant change in ALS or any proteins involved in amino acid synthesis in leaves and developing anthers of the MES-treated plants was identified in this study, although ALS is the target for herbicides sulphonylurea family [13]. Furthermore, the change in *ALS* transcripts was not detected when genome-wide expression profiling was conducted by the microarray technique using the same set of samples as in the present study (unpublished). Similar results have been reported on genome-wide expression profiling based on Affymetrix ATH1 arrays in *A. thaliana* and *B. napus* leaves after sulphonylurea treatment [14]. These findings suggested that ALS-inhibitor treatment depresses the activity of ALS but does not affect its expression. The mRNA level is not always well correlated with the protein level mainly because of post-transcriptional regulation such as mRNA processing, transcript stability, translational regulation and protein degradation [49], [50]. However, comparison of our proteomic data with transcriptional data of sulphonylurea family members acting as herbicides in *Arabidopsis* leaves may help elucidate the biochemical and molecular mechanisms by which CHA-MES treatment induces plant male sterility [51]–[53]. Transcripts involved in metabolism, RNA synthesis, protein synthesis, DNA replication, stress, cell wall synthesis, hormone signalling, development, transport and photosynthesis are reportedly affected in *Arabidopsis* leaves after herbicide sulphonylurea treatment with a concentration of 0.131 g ha^−1^ active ingredient (sulphometuron methyl), leading to a 50% reduction in shoot dry weight [14]. However, in the present investigation, application of low-dosage MES (1% of the concentration required for its herbicidal effect) as CHA to *B. napus* only induced minimal changes such as stress responses, protein metabolism and photosynthesis in leaves. Moreover, the vegetable tissues of MES-treated plants normally grew without apparent phenotypic alteration. By contrast, in developing anthers that are the most sensitive tissues in plants, the abundances of many proteins involved in hormone signalling, transcription, protein, lipid and DNA biosynthesis, plant development/differentiation, cytoskeleton dynamics, cell wall remodelling/metabolism as well as intracellular trafficking were down-regulated after MES treatment. These changes consequently affected the development of anthers and generated defective tapetum and abnormal microspores throughout all anther development stages, thereby resulting in male sterility in *B. napus*. Thus, low-dose CHA-MES treatment induced obvious changes in the proteome of developing anthers, resulting in abnormal tapetum and microspores at the entire anther development stages and ultimately in male sterility. However, the normal growth of vegetable tissues was unaffected probably because of their high tolerance to such a minor environmental stress.

In summary, cytological and proteomic changes in leaves, small buds, anthers from medium buds and anthers from large buds of male sterile rapeseed plants induced by CHA-MES were investigated. The cytological study indicated that rapeseed male sterility induced by MES-treatment may be mainly due to the gradually generated abnormal tapetum and defective microspores in developing anthers of MES-treated plants at various anther development stages. A total of 141 differentially expressed proteins were revealed, including 9 in leaves, 8 in small buds, 24 in anthers from medium buds and 100 in anthers from large buds. Amongst these 141 proteins, 131 were further identified by MALDI-TOF/TOF MS and found to be mainly involved in several processes that may be crucial to tapetum and microspore development. The down-regulation of these proteins may disrupt the coordination of developmental and metabolic processes, resulting in defective tapetal cells and abnormal microspores that led to male sterility in MES-treated plants. Accordingly, a simple model of CHA-MES-induced male sterility in *B. napus* was established. To the best of our knowledge, this is the first cytological and dynamic proteomic investigation on the mechanism by which CHAs from the sulphonylurea family induce male sterility in plants. The results expand knowledge on the complexity of anther proteins in male sterile *B. napus*, and provide a framework for further functional studies on each identified protein.

## Supporting Information

Dataset S1 **Annotated spectra for all of the 131 differentially expressed proteins identified by PMF.**
(DOC)

Figure S1 **Ultrastructure of microspores and tapetal cells of the normal fertile anthers from control plants and abnormal anthers from the Mes-treated plants throughout stages of pollen development.** (A) the fertile microspores of control plants at pollen mother cell stage showing vacuoles and plastids. (B) the fertile microspores of control plants at tetrad stage showing abundant organells. (C) the fertile microspores of control plants at middle-microspore stage showing abundant organells. (D) the fertile microspores of control plants at vacuolated-microspore stage showing large vacuole and many other properly organized organells. (E) the sterile microspores of the Mes-induced male sterile anther at pollen mother cell stage showing vacuoles and plastids but plasma membrane segregated from cell wall seriously. (F) the sterile microspores of the Mes-induced male sterile anther at tetrad stage showing a few organells. (G) the sterile microspores of the Mes-induced male sterile anther at middle-microspore stage showing cell debris in the center. (H) the sterile microspores of the Mes-induced male sterile anther at vacuolated microspore stage showing cell debris in the center. (I) the fertile tapetal cells of control plants at pollen mother cell stage showing vacuoles and other various organells. (J) the fertile tapetal cells at tetrad stage showing abundant organells. (K) the fertile tapetal cells at middle-microspore stage showing abundant organells. (L) the fertile tapetal cells at vacuolated-microspore stage contained many plastids with plenty of starch granules. (M–P) the sterile tapetal cells of the Mes-induced male sterile anther at pollen mother cell, tetrad, middle-microspore and-vacuolated microspore stage respectively containing cytoplasms with much less volume and few organelles. (Q) fertile mature pollen of control plants. (R–T) sterile pollen of the Mes-induced male sterile plants. Note: m, mitochondria; ga, Golgi; ex, exine; pl, plastid; v, vacuole; er, endoplasmic reticulum.
(TIF)

Figure S2 **Representative 2-DE gels of proteins in leaves, small buds, anthers from medium buds and anthers from large buds of control and MES-treated plants.** Total proteins were extracted by the TCA–acetone precipitation method and separated by IEF/SDS-PAGE. Proteins were stained with Coomassie Brilliant Blue G-250. Protein samples (800 µg) were loaded onto pH 4–7 IPG strips (17 cm, linear). SDS-PAGE was performed with 11% gels. A total of 9 differentially expressed protein spots in leaves of control (A) and MES-treated (B) plants, 8 differentially expressed protein spots in small buds of control (C) and MES-treated (D) plants, 24 differentially expressed protein spots in anthers from medium buds of control (E) and MES-treated (F) plants and 100 differentially expressed protein spots in anthers from large buds of control (G) and MES-treated (H) plants are numbered. The protein spot numbers correspond to Table S2.
(TIF)

Table S1 **Differentially expressed protein spots in tissues between the Mes-treated and the control plants.**
(DOC)

Table S2 **Identities of differentially expressed proteins by PMF analysis.**
(DOC)

Table S3 **Corresponding homologues of the 23 unknown proteins.**
(DOC)
