# Supplementary material for: Cytological and Comparative Proteomic Analyses on Male Sterility in Brassica napus L. Induced by the Chemical Hybridization Agent Monosulphuron Ester Sodium
Source: PLoS One. 2013 Nov 14;8(11):e80191. doi: 10.1371/journal.pone.0080191 (PMC3828188; doi:10.1371/journal.pone.0080191)
Supplement: Dataset S1 — Annotated spectra for all of the 131 differentially expressed proteins identified by PMF. (DOC) [file pone.0080191.s001.doc]

**Dataset S1. Annotated spectra for all of the 131 differentially expressed proteins identified by PMF.**

Spot numbers of the 131 proteins correspond to that list in Table S2.

Spot No.: **1**

Accession No.: gi|54036487

Score: 73

Spot No.:**2**

Accession No.: gi|55701025

Score: 80

Spot No.:**3**

Accession No.: gi|74273101

Score: 72

Spot No.:**4**

Accession No.: gi|2204102

Score: 105

Spot No.:**5**

Accession No.: gi|2792222

Score: 78

Spot No.:**6**

Accession No.: gi|170177802

Score: 176

Spot No.:**7**

Accession No.: gi|49359169

Score: 75

Spot No.:**8**

Accession No.: gi|21537296

Score: 76

Spot No.:**9**

Accession No.: gi|297795029

Score: 74

Spot No.:**11**

Accession No.: gi|240255605

Score: 83

Spot No.:**12**

Accession No.: gi|51535085

Score: 85

Spot No.:**13**

Accession No.: gi|11135407

Score: 80

Spot No.:**14**

Accession No.: gi|297819272

Score: 258

Spot No.:**15**

Accession No.: gi|2599092

Score: 86

Spot No.:**16**

Accession No.: gi|8885622

Score: 103

Spot No.:**17**

Accession No.: gi|77554545

Score: 101

Spot No.: **18**

Accession No.: gi|304325130

Score:75

Spot No.: **19**

Accession No.: gi|297819272

Score: 92

Spot No.: **20**

Accession No.: gi|145362282

Score: 116

Spot No.: **21**

Accession No.: gi|116779313

Score: 76

Spot No.: **22**

Accession No.: gi|15237362

Score: 120

Spot No.: **23**

Accession No.: gi|312231793

Score: 80

Spot No.: **24**

Accession No.: gi|297803740

Score: 103

Spot No.: **25**

Accession No.: gi|125972284

Score: 72

Spot No.: **26**

Accession No.: gi|112490556

Score: 184

Spot No.: **27**

Accession No.: [gi|62126055](http://www.matrixscience.com/cgi/protein_view.pl?file=../data/20110128/FttcIeunt.dat&hit=1)

Score: 72

Spot No.: **28**

Accession No.: gi|294845743

Score: 164

Spot No.: **29**

Accession No.: gi|159476424

Score: 79

Spot No.: **31**

Accession No.: gi|30580468

Score: 78

Spot No.: **32**

Accession No.: gi|132270

Score: 74

Spot No.: **33**

Accession No.: [gi|30688506](http://www.matrixscience.com/cgi/protein_view.pl?file=../data/20110128/FttcIecmL.dat&hit=2)

Score: 99

Spot No.: **34**

Accession No.: [gi|30688506](http://www.matrixscience.com/cgi/protein_view.pl?file=../data/20110128/FttcIecnS.dat&hit=2)

Score: 76

Spot No.: **35**

Accession No.: [gi|30688506](http://www.matrixscience.com/cgi/protein_view.pl?file=../data/20110128/FttcIecnS.dat&hit=2)

Score: 99

Spot No.: **36**

Accession No.: gi|297792679

Score: 89

Spot No.: **37**

Accession No.: gi|145355325

Score: 73

Spot No.: **38**

Accession No.: gi|18415850

Score: 75

Spot No.: **39**

Accession No.: gi|168062532

Score: 72

Spot No.: **40**

Accession No.: gi|224138342

Score: 77

Spot No.: **41**

Accession No.: gi|303283614

Score: 74

Spot No.: **42**

Accession No.: gi|22331535

Score: 100

Spot No.: **43**

Accession No.: gi|22331535

Score: 93

Spot No.: **44**

Accession No.: gi|25090053

Score: 119

Spot No.: **45**

Accession No.: gi|25090053

Score: 119

Spot No.: **46**

Accession No.: gi|119655911

Score: 89

Spot No.: **47**

Accession No.: gi|18404382

Score: 107

Spot No.: **48**

Accession No.: gi|297819782

Score: 110

Spot No.: **49**

Accession No.: gi|15228667

Score: 104

Spot No.: **50**

Accession No.: gi|18405145

Score: 80

Spot No.: **51**

Accession No.: gi|18405145

Score: 80

Spot No.: **52**

Accession No.: gi|266533

Score: 94

Spot No.: **53**

Accession No.: gi|13124444

Score: 215

Spot No.: **54**

Accession No.: gi|15242717

Score: 113

Spot No.: **55**

Accession No.: gi|55296320

Score: 77

Spot No.: **56**

Accession No.: gi|6624302

Score: 72

Spot No.: **58**

Accession No.: gi|297795465

Score: 85

Spot No.: **59**

Accession No.: gi|159479650

Score: 75

Spot No.: **60**

Accession No.: gi|19439

Score: 75

Spot No.: **61**

Accession No.: gi|303276555

Score: 80

Spot No.: **63**

Accession No.: [gi|113205339](http://www.matrixscience.com/cgi/protein_view.pl?file=../data/20110314/Fttmlaeae.dat&hit=1)

Score: 80

Spot No.: **64**

Accession No.: gi|224071423

Score: 80

Spot No.: **65**

Accession No.: gi|75309952

Score: 94

Spot No.: **66**

Accession No.: gi|29839389

Score: 141

Spot No.: **67**

Accession No.: gi|4928472

Score: 85

Spot No.: **68**

Accession No.: gi|15241704

Score: 97

Spot No.: **69**

Accession No.: gi|15241704

Score: 124

Spot No.: **70**

Accession No.: gi|15241704

Score: 130

Spot No.: **71**

Accession No.: gi|15242316

Score: 152

Spot No.: **72**

Accession No.: gi|15232865

Score: 95

Spot No.: **73**

Accession No.: gi|15242351

Score: 164

Spot No.: **75**

Accession No.: gi|21537260

Score: 106

Spot No.: **76**

Accession No.: gi|21537260

Score: 92

Spot No.: **77**

Accession No.: gi|79470337

Score: 80

Spot No.: **78**

Accession No.: gi|18265381

Score: 154

Spot No.: **79**

Accession No.: gi|297824281

Score: 217

Spot No.: **80**

Accession No.: gi|77999357

Score: 91

Spot No.: **81**

Accession No.: gi|77999357

Score: 267

Spot No.: **82**

Accession No.: gi|297839799

Score: 105

Spot No.: **83**

Accession No.: gi|110736416

Score: 88

Spot No.: **84**

Accession No.: gi|15226453

Score: 79

Spot No.: **85**

Accession No.: gi|15226453

Score: 87

Spot No.: **86**

Accession No.: gi|15229530

Score: 80

Spot No.: **87**

Accession No.: gi|15229530

Score: 80

Spot No.: **88**

Accession No.: gi|270054998

Score: 100

Spot No.: **89**

Accession No.: gi|15231176

Score: 86

Spot No.: **90**

Accession No.: gi|30693102

Score: 177

Spot No.: **91**

Accession No.: gi|11869927

Score: 77

Spot No.: **92**

Accession No.: gi|86769414

Score: 75

Spot No.: **93**

Accession No.: gi|14422255

Score: 161

Spot No.: **94**

Accession No.: gi|15242717

Score: 86

Spot No.: **95**

Accession No.: gi|30690246

Score: 109

Spot No.: **96**

Accession No.: gi|15232763

Score: 88

Spot No.: **97**

Accession No.: gi|15242717

Score: 107

Spot No.: **98**

Accession No.: gi|159472581

Score: 78

Spot No.: **99**

Accession No.: gi|15239020

Score: 81

Spot No.: **100**

Accession No.: gi|91694371

Score: 75

Spot No.: **101**

Accession No.: gi|98991380

Score: 99

Spot No.: **102**

Accession No.: gi|168005127

Score: 80

Spot No.: **103**

Accession No.: gi|168005127

Score: 80

Spot No.: **104**

Accession No.: gi|18394249

Score: 78

Spot No.: **105**

Accession No.: gi|81176557

Score: 313

Spot No.: **106**

Accession No.: gi|115345735

Score: 228

Spot No.: **107**

Accession No.: gi|301751677

Score: 78

Spot No.: **108**

Accession No.: gi|42409328

Score: 88

Spot No.: **109**

Accession No.: gi|62902938

Score: 77

Spot No.: **110**

Accession No.: gi|45775570

Score: 83

Spot No.: **111**

Accession No.: gi|255080776

Score: 73

Spot No.: **112**

Accession No.: gi|297793857

Score: 75

Spot No.: **113**

Accession No.: gi|18409908

Score: 96

Spot No.: **114**

Accession No.: gi|166582

Score: 205

Spot No.: **115**

Accession No.: gi|15231447

Score: 193

Spot No.: **116**

Accession No.: gi|166582

Score: 89

Spot No.: **117**

Accession No.: gi|159467825

Score: 83

Spot No.: **118**

Accession No.: gi|159476658

Score: 74

Spot No.: **120**

Accession No.: gi|297832912

Score: 101

Spot No.: **121**

Accession No.: gi|255552951

Score: 77

Spot No.: **122**

Accession No.: gi|297740015

Score: 78

Spot No.: **123**

Accession No.: gi|225447009

Score: 73

Spot No.: **124**

Accession No.: gi|168061841

Score: 80

Spot No.: **125**

Accession No.: gi|242055697

Score: 82

Spot No.: **126**

Accession No.: gi|21741358

Score: 80

Spot No.: **127**

Accession No.: gi|168021219

Score: 81

Spot No.: **128**

Accession No.: gi|115461226

Score: 80

Spot No.: **129**

Accession No.: gi|242036443

Score: 77

Spot No.: **130**

Accession No.: gi|297811689

Score: 102

Spot No.: **131**

Accession No.: gi|115444219

Score: 74
