# Supplementary figures and images for: Cytological and Comparative Proteomic Analyses on Male Sterility in Brassica napus L. Induced by the Chemical Hybridization Agent Monosulphuron Ester Sodium
Source: PLoS One. 2013 Nov 14;8(11):e80191. doi: 10.1371/journal.pone.0080191 (PMC3828188; doi:10.1371/journal.pone.0080191)

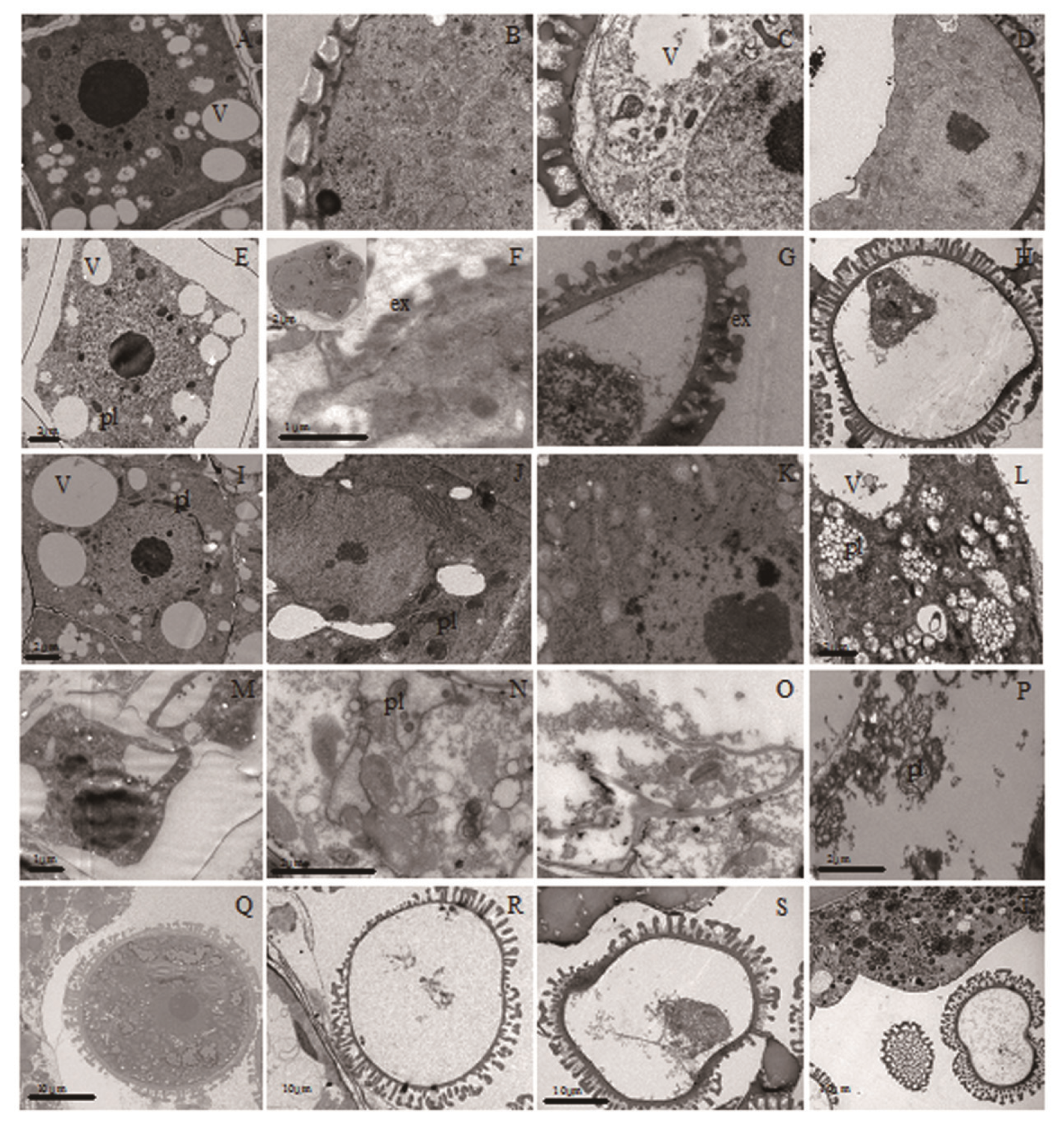

Supplement: Figure S1 — Ultrastructure of microspores and tapetal cells of the normal fertile anthers from control plants and abnormal anthers from the Mes-treated plants throughout stages of pollen development. (A) the fertile microspores of control plants at pollen mother cell stage showing vacuoles and plastids. (B) the fertile microspores of control plants at tetrad stage showing abundant organells. (C) the fertile microspores of control plants at middle-microspore stage showing abundant organells. (D) the fertile microspores of control plants at vacuolated-microspore stage showing large vacuole and many other properly organized organells. (E) the sterile microspores of the Mes-induced male sterile anther at pollen mother cell stage showing vacuoles and plastids but plasma membrane segregated from cell wall seriously. (F) the sterile microspores of the Mes-induced male sterile anther at tetrad stage showing a few organells. (G) the sterile microspores of the Mes-induced male sterile anther at middle-microspore stage showing cell debris in the center. (H) the sterile microspores of the Mes-induced male sterile anther at vacuolated microspore stage showing cell debris in the center. (I) the fertile tapetal cells of control plants at pollen mother cell stage showing vacuoles and other various organells. (J) the fertile tapetal cells at tetrad stage showing abundant organells. (K) the fertile tapetal cells at middle-microspore stage showing abundant organells. (L) the fertile tapetal cells at vacuolated-microspore stage contained many plastids with plenty of starch granules. (M–P) the sterile tapetal cells of the Mes-induced male sterile anther at pollen mother cell, tetrad, middle-microspore and-vacuolated microspore stage respectively containing cytoplasms with much less volume and few organelles. (Q) fertile mature pollen of control plants. (R–T) sterile pollen of the Mes-induced male sterile plants. Note: m, mitochondria; ga, Golgi; ex, exine; pl, plastid; v, vacuole; er, endoplasmic r [file pone.0080191.s002.tif]

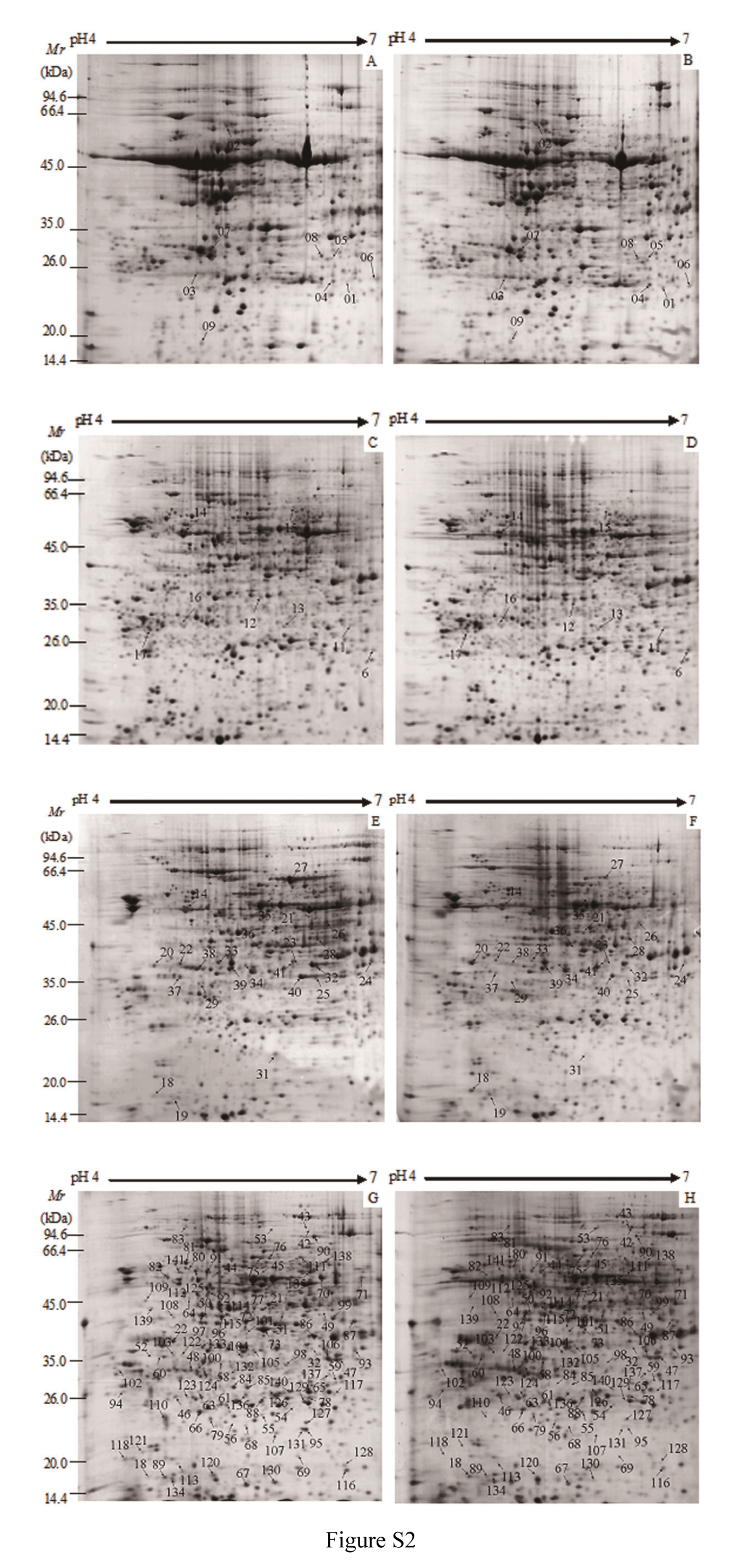

Supplement: Figure S2 — Representative 2-DE gels of proteins in leaves, small buds, anthers from medium buds and anthers from large buds of control and MES-treated plants. Total proteins were extracted by the TCA–acetone precipitation method and separated by IEF/SDS-PAGE. Proteins were stained with Coomassie Brilliant Blue G-250. Protein samples (800 µg) were loaded onto pH 4–7 IPG strips (17 cm, linear). SDS-PAGE was performed with 11% gels. A total of 9 differentially expressed protein spots in leaves of control (A) and MES-treated (B) plants, 8 differentially expressed protein spots in small buds of control (C) and MES-treated (D) plants, 24 differentially expressed protein spots in anthers from medium buds of control (E) and MES-treated (F) plants and 100 differentially expressed protein spots in anthers from large buds of control (G) and MES-treated (H) plants are numbered. The protein spot numbers correspond to Table S2. (TIF) [file pone.0080191.s003.tif]
