# Supplementary material for: Cytological and Comparative Proteomic Analyses on Male Sterility in Brassica napus L. Induced by the Chemical Hybridization Agent Monosulphuron Ester Sodium
Source: PLoS One. 2013 Nov 14;8(11):e80191. doi: 10.1371/journal.pone.0080191 (PMC3828188; doi:10.1371/journal.pone.0080191)
Supplement: Table S1 — Differentially expressed protein spots in tissues between the Mes-treated and the control plants. (DOC) [file pone.0080191.s004.doc]

Table S1 Differentially expressed protein spots in tissues between the Mes-treated and the control plants

| Spot No. | Spot intensity (Mean±SD) ^a^ | | Fold change^b^ | Tissue^c^ |
| --- | --- | --- | --- | --- |
|  | Control | Treatment |  |  |
| 01 | 3920.1±520.4 | 8934.5±675.1 | +2.28﹡﹡ | Ls |
| 02 | 9007.7±1103.5 | 18595.1±3506.6 | +2.06﹡ | Ls |
| 03 | 8352.9±960.7 | 17157.8±2083.3 | +2.05﹡ | Ls |
| 04 | 11176.7±1228.0 | 16813.9±825.9 | +1.50﹡ | Ls |
| 05 | 4766.7±658.9 | 8210.3±83.2 | +1.72﹡ | Ls |
| 06 | 3710.1±241.5 | 13383.4±1636.6 | +3.61﹡ | Ls |
| 06 | 8657.7±467.2 | 16745.9±2398.2 | +1.93﹡ | SBs |
| 07 | 73176.6±4343.5 | 37732.5±3509.2 | -1.94﹡ | Ls |
| 08 | 22255.7±3322.0 | 12336.4±1347.6 | -1.80﹡ | Ls |
| 09 | 6260.1±675.2 | 4169.3±1588.7 | -1.50* | Ls |
| 11 | 9385.2±987.0 | 15760.6±1764.6 | +1.68﹡ | SBs |
| 12 | 16523.3±1307.7 | 24806.8±1145.5 | +1.50﹡ | SBs |
| 13 | 15210.8±668.9 | 10133.43±808.57 | -1.50﹡﹡ | SBs |
| 14 | 88354.4±3598.1 | 43926.7±2851.2 | -2.01﹡ | SBs |
| 14 | 64623.0±4193.4 | 41715.9±6818.7 | -1.55﹡﹡ | An-MBs |
| 15 | 21856.2±1744.0 | 11224.2±1245.9 | -1.95﹡ | SBs |
| 16 | 13524.6±1259.8 | 7157.3±1109.3 | -1.89﹡ | SBs |
| 17 | 15724.4±114.9 | 9085.8±511.0 | -1.73﹡﹡ | SBs |
| 18 | 30071.6±6907.7 | 63966.0±10579.1 | +2.13﹡ | An-MBs |
| 18 | 13212.2±383.4 | 36399.4±513.7 | +2.76﹡﹡ | An-LBs |
| 19 | 23780.3±5108.8 | 55488.5±7152.9 | +2.33﹡ | An-MBs |
| 20 | 48099.3±8288.0 | 122609.0±22732.3 | +2.55﹡ | An-MBs |
| 21 | 45160.7±5819.9 | 24219.1±2028.2 | +1.88﹡ | An-MBs |
| 21 | 25180.2±2981.4 | 42791.0±225.6 | +1.70﹡ | An-LBs |
| 22 | 49147.8±2036.8 | 27532.8±5277.2 | -1.79﹡ | An-MBs |
| 22 | 55838.1±3887.1 | 8124.7±32.1 | -6.87﹡﹡ | An-LBs |
| 23 | 16991.1±2204.8 | 0 | - | An-MBs |
| 24 | 57651.7±7940.0 | 29989.8±4683.9 | -1.92﹡﹡ | An-MBs |
| 25 | 23605.1±8081.1 | 0 | - | An-MBs |
| 26 | 54936.9±5764.4 | 31753.9±1213.7 | -1.73﹡ | An-MBs |
| 27 | 320635.9±53021.6 | 54922.3±9541.1 | -6.22﹡﹡ | An-MBs |
| 28 | 89270.2±8359.3 | 44296.8±6368.2 | -2.02﹡ | An-MBs |
| 29 | 11513.5±5455.7 | 0 | - | An-MBs |
| 31 | 41281.1±15627.3 | 0 | - | An-MBs |
| 32 | 32796.8±1891.1 | 13600.0±1665.9 | -2.41﹡﹡ | An-MBs |
| 32 | 10146.8±367.5 | 0 | - | An-LBs |
| 33 | 53991.8±3131.1 | 23588.3±4109.1 | -2.29﹡﹡ | An-MBs |
| 34 | 31392.9±6158.5 | 11765.6±1754.7 | -2.67﹡ | An-MBs |
| 35 | 23375.4±3834.6 | 9446.9±245.0 | -2.47﹡ | An-MBs |
| 36 | 19111.4±3489.4 | 0 | - | An-MBs |
| 37 | 112904.0±12072.4 | 25243.1±2631.7 | -4.47﹡ | An-MBs |
| 38 | 95326.6±4134.3 | 14837.3±497.1 | -6.42﹡ | An-MBs |
| 39 | 34339.5±1330.0 | 21164.6±2737.0 | -1.62﹡ | An-MBs |
| 40 | 155596.2±18345.7 | 40517.8±2740.8 | -3.83﹡ | An-MBs |
| 41 | 37550.8±4553.0 | 16798.0±5417.7 | -2.24﹡ | An-MBs |
| 42 | 0 | 61776.2±1550.9 | + | An-LBs |
| 43 | 0 | 50366.0±1899.8 | + | An-LBs |
| 44 | 11112.3±246.1 | 20254.5±421.8 | +1.82﹡ | An-LBs |
| 45 | 5070.8±394.4 | 10007.1±337.9 | +1.97﹡﹡ | An-LBs |
| 46 | 21924.9±1380.0 | 57909.6±3126.5 | +2.64﹡﹡ | An-LBs |
| 47 | 20739.8±488.7 | 10079.0±151.0 | +1.61﹡﹡ | An-LBs |
| 48 | 30255.7±267.4 | 54369.7±4408.3 | +2.01﹡ | An-LBs |
| 49 | 0 | 28313.7±6526.1 | + | An-LBs |
| 50 | 9277.1±231.2 | 19719.6±251.9 | +2.13﹡﹡ | An-LBs |
| 51 | 11710.4±1401.4 | 32422.5±3662.0 | +2.77﹡ | An-LBs |
| 52 | 0 | 44351.0±4824.8 | + | An-LBs |
| 53 | 0 | 32399.3±2663.9 | + | An-LBs |
| 54 | 0 | 32162.4±6958.5 | + | An-LBs |
| 55 | 10611.7±451.7 | 18935.6±548.9 | +1.78﹡﹡ | An-LBs |
| 56 | 0 | 16254.4±455.1 | + | An-LBs |
| 58 | 13060.2±830.7 | 42790.7±225.6 | +3.65﹡﹡ | An-LBs |
| 59 | 0 | 10587.2±228.0 | + | An-LBs |
| 60 | 0 | 15485.5±514.6 | + | An-LBs |
| 61 | 12127.3±683.6 | 27370.0±698.6 | +2.26﹡﹡ | An-LBs |
| 63 | 5942.8±315.2 | 0 | - | An-LBs |
| 64 | 9284.8±996.6 | 0 | - | An-LBs |
| 65 | 11656.0±617.6 | 0 | - | An-LBs |
| 66 | 75327.1±2042.1 | 42984.7±2854.1 | -1.75﹡﹡ | An-LBs |
| 67 | 70376.4±4227.0 | 30977.0±2353.5 | -2.27﹡﹡ | An-LBs |
| 68 | 7246.6±457.8 | 0 | - | An-LBs |
| 69 | 22140.4±4039.4 | 0 | - | An-LBs |
| 70 | 131082.6±6996.4 | 30315.5±1431.1 | -4.34﹡ | An-LBs |
| 71 | 34657.2±471.2 | 5452.5±514.4 | -6.36﹡﹡ | An-LBs |
| 72 | 24157.5±744.2 | 0 | - | An-LBs |
| 73 | 214924.2±11273.3 | 71316.4±2163.8 | -3.01﹡﹡ | An-LBs |
| 75 | 25067.5±510.3 | 11922.0±2033.8 | -2.10﹡﹡ | An-LBs |
| 76 | 20456.0±782.8 | 8911.2±1189.3 | -2.30﹡﹡ | An-LBs |
| 77 | 12768.2±1693.7 | 0 | - | An-LBs |
| 78 | 45587.3±3187.2 | 24054.6±1436.4 | -1.90﹡ | An-LBs |
| 79 | 27318.5±945.2 | 11373.6±1447.7 | -2.40﹡﹡ | An-LBs |
| 80 | 11340.6±366.9 | 0 | - | An-LBs |
| 81 | 63575.7±1324.2 | 37123.5±3268.2 | -1.71﹡﹡ | An-LBs |
| 82 | 35012.0±2567.5 | 0 | - | An-LBs |
| 83 | 10591.4±488.8 | 0 | - | An-LBs |
| 84 | 45896.9±2379.4 | 0 | - | An-LBs |
| 85 | 35145.0±4412.0 | 0 | - | An-LBs |
| 86 | 103997.9±10690.8 | 40315.5±3279.4 | -2.58﹡ | An-LBs |
| 87 | 61529.7±4166.4 | 16589.0±2596.3 | -3.71﹡﹡ | An-LBs |
| 88 | 14715.4±297.5 | 6758.8±357.0 | -2.18﹡﹡ | An-LBs |
| 89 | 45775.4±2430.2 | 18765.4±3467.2 | -2.44﹡﹡ | An-LBs |
| 90 | 44947.2±1895.2 | 20173.5±4085.6 | -2.23﹡ | An-LBs |
| 91 | 54846.3±3649.2 | 14466.0±353.5 | -3.79﹡﹡ | An-LBs |
| 92 | 23305.1±1390.3 | 0 | - | An-LBs |
| 93 | 38891.7±4492.6 | 17847.1±1279.4 | -2.18﹡ | An-LBs |
| 94 | 15644.6±390.9 | 0 | - | An-LBs |
| 95 | 51481.8±1482.0 | 4899.4±63.8 | -10.58﹡﹡ | An-LBs |
| 96 | 75378.7±192.9 | 45282.4±2409.8 | -1.66﹡﹡ | An-LBs |
| 97 | 123311.0±9358.7 | 71739.5±479.4 | -1.72﹡ | An-LBs |
| 98 | 18906.2±1079.6 | 0 | - | An-LBs |
| 99 | 47338.8±3692.93 | 18319.27±2614.49 | -2.58±0.59﹡ | An-LBs |
| 100 | 47338.8±3692.9 | 0 | - | An-LBs |
| 101 | 35867.7±3104.5 | 0 | - | An-LBs |
| 102 | 8011.2±334.4 | 0 | - | An-LBs |
| 103 | 13339.7±399.7 | 0 | - | An-LBs |
| 104 | 20201.6±1027.5 | 0 | - | An-LBs |
| 105 | 17609.3±112.8 | 9244.6±1279.3 | -1.90﹡ | An-LBs |
| 106 | 80828.6±9352.5 | 13564.1±2160.2 | -5.96﹡﹡ | An-LBs |
| 107 | 17451.2±1215.8 | 0 | - | An-LBs |
| 108 | 9804.7±997.3 | 0 | - | An-LBs |
| 109 | 14994.1±1398.1 | 0 | - | An-LBs |
| 110 | 133782.4±7200.5 | 0 | - | An-LBs |
| 111 | 18383.9±1078.9 | 0 | - | An-LBs |
| 112 | 5669.7±945.1 | 0 | - | An-LBs |
| 113 | 11687.3±1206.8 | 5515.8±496.9 | -2.12﹡ | An-LBs |
| 114 | 119039.7±8206.4 | 12179.2±485.2 | -9.77﹡ | An-LBs |
| 115 | 91386.8±5240.3 | 38279.4±6444.8 | -2.39﹡ | An-LBs |
| 116 | 45468.4±892.7 | 20507.4±3030.0 | -2.22﹡﹡ | An-LBs |
| 117 | 16781.6±1972.6 | 0 | - | An-LBs |
| 118 | 35992.7±5658.9 | 0 | - | An-LBs |
| 120 | 61606.6±38.5 | 0 | - | An-LBs |
| 121 | 27227.2±3523.3 | 0 | - | An-LBs |
| 122 | 5630.2±107.2 | 0 | - | An-LBs |
| 123 | 10582.0±1298.4 | 0 | - | An-LBs |
| 124 | 30658.2±26.8 | 0 | - | An-LBs |
| 125 | 31640.8±1800.3 | 0 | - | An-LBs |
| 126 | 28647.0±2921.6 | 0 | - | An-LBs |
| 127 | 30705.5±2460.1 | 12455.2±164.1 | -2.47﹡﹡ | An-LBs |
| 128 | 27482.2±3055.9 | 17091.1±1224.8 | -1.61﹡ | An-LBs |
| 129 | 26313.7±4167.6 | 0 | - | An-LBs |
| 130 | 67294.2±4347.8 | 4933.0±79.4 | -13.64﹡ | An-LBs |
| 131 | 19121.1±1685.6 | 3782.2±219.8 | -5.54﹡ | An-LBs |
| 132 | 0 | 21258.3±1671.3 | + | An-LBs |
| 133 | 40924.9±7645.8 | 87686.1±6026.7 | +2.25﹡ | An-LBs |
| 134 | 20752.0±912.9 | 0 | - | An-LBs |
| 135 | 25736.0±1662.4 | 9446.0±472.0 | -2.72﹡ | An-LBs |
| 136 | 24476.4±4645.0 | 10003.5±985.5 | -2.45﹡ | An-LBs |
| 137 | 36747.5±2071.7 | 0 | - | An-LBs |
| 138 | 23672.5±1010.5 | 0 | - | An-LBs |
| 139 | 30607.0±1291.9 | 0 | - | An-LBs |
| 140 | 36124.3±1458.0 | 13211.2±169.2 | -2.73﹡ | An-LBs |
| 141 | 54946.5±6747.3 | 0 | - | An-LBs |

^a^ Spot intensity is the mean value of three biological replicates of each spot ± standard deviation.

^b^ Spot abundance is expressed as the ratio of intensities of up-regulated (plus value) or down-regulated (minus value) proteins between Mes-treatment and control. -Fold changes had p values, “*” represents p<0.05; “**” represents p<0.01. “-” represents protein spots detected only in the control plant tissues but absent in Mes-treated plant tissues, “+” represents protein spots detected only in Mes-treated tissues but not in control plants.

^C^ “Ls” represents leaves, “SBs” represents small buds in length less than 1 mm, “An-MBs” represents anthers from medium buds of 1～3 mm in length (from meiosis to vacuolated-microspore stage), and “An-LBs” represents anthers from large buds with length longer than 3 mm (from vocuolated-microspore to mature pollen stage).
