# Supplementary material for: Cytological and Comparative Proteomic Analyses on Male Sterility in Brassica napus L. Induced by the Chemical Hybridization Agent Monosulphuron Ester Sodium
Source: PLoS One. 2013 Nov 14;8(11):e80191. doi: 10.1371/journal.pone.0080191 (PMC3828188; doi:10.1371/journal.pone.0080191)
Supplement: Table S2 — Identities of differentially expressed proteins by PMF analysis. (DOC) [file pone.0080191.s005.doc]

**Table S2.** Identities of differentially expressed proteins by PMF analysis

| Spot no. | | Gene ID in NCBI | Protein name | Theoretical/observed | | Score | M ^a^ | | C ^b^ | | Fold change ^c^ | Stage ^d^ |
| --- | --- | --- | --- | --- | --- | --- | --- | --- | --- | --- | --- | --- |
|  |  |  |  | *M*_r_ (kDa) | pI |  |  |  |  |  |  |  |
| **Up-regulated** | | | | | | | | | | | | |
| **Cell rescue, defence and virulence** | | | | | | | | | | | | |
| 02 | | 55701025 | TPA: class III peroxidase 79 precursor | 41.1/64.3 | 5.94/5.33 | 80 | 12 | | 32 | | +2.06** | Ls |
| 04 | | 2204102 | Glutathione-S-transferase | 15.5/25.1 | 5.41/6.34 | 105 | 14 | | 49 | | +1.50* | Ls |
| 05 | | 2792222 | NBS-LRR type resistance protein | 36.1/30.8 | 9.01/6.33 | 78 | 9 | | 29 | | +1.72* | Ls |
| 06 | | 170177802 | Glutathione S-transferase | 22.6/25.0 | 5.81/6.70 | 176 | 15 | | 62 | | +3.61* | Ls |
| 06 | | 170177802 | Glutathione S-transferase | 22.6/25.0 | 5.81/6.70 | 176 | 15 | | 62 | | +1.93* | SBs |
| 18 | | 304325130 | Rp1-like protein | 141.7/18.1 | 6.31/4.72 | 75 | 21 | | 20 | | +2.13* | An-MBs |
| 18 | | 304325130 | Rp1-like protein | 141.7/17.4 | 6.31/4.68 | 75 | 21 | | 20 | | +2.76** | An-LBs |
| **Carbohydrate Metabolism** | | | | | | | | | | | | |
| 42 | | 22331535 | UDP-glycosyltransferase/ sucrose synthase/ transferase, transferring glycosyl groups | 93.5/95.9 | 6.12/6.12 | 100 | 22 | | 25 | | + | An-LBs |
| 43 | | 22331535 | UDP-glycosyltransferase/ sucrose synthase/ transferase, transferring glycosyl groups | 93.5/95.9 | 6.12/6.09 | 93 | 14 | | 18 | | + | An-LBs |
| 44 | | 25090053 | Aldehyde dehydrogenase family 7 member A1 | 53.2/55.8 | 5.52/5.31 | 119 | 15 | | 34 | | +1.82* | An-LBs |
| 45 | | 25090053 | Aldehyde dehydrogenase family 7 member A1 | 53.2/55.8 | 5.52/5.71 | 119 | 15 | | 34 | | +1.97** | An-LBs |
| 46 | | 119655911 | Thiol methyltransferase | 25.3/28.0 | 4.90/4.95 | 89 | 8 | | 46 | | +2.64** | An-LBs |
| 47 | | 18404382 | Malate dehydrogenase (NAD), mitochondrial | 36.0/36.4 | 8.54/6.37 | 107 | 12 | | 45 | | +1.61** | An-LBs |
| **Energy Metabolism** | | | | | | | | | | | | |
| 48 | | 297819782 | Photosystem II subunit O-2 | 35.3/33.6 | 5.93/5.10 | 110 | 14 | | 56 | | +2.01* | An-LBs |
| 49 | | 15228667 | GDH3 (glutamate dehydrogenase 3) | 44.8/43.5 | 5.75/6.23 | 104 | 14 | | 29 | | + | An-LBs |
| 50 | | 18405145 | RCA (rubisco activase) | 52.4/42.9 | 5.87/5.24 | 80 | 10 | | 18 | | +2.13** | An-LBs |
| 51 | | 18405145 | RCA (rubisco activase) | 52.4/43.4 | 5.87/5.90 | 80 | 10 | | 18 | | +2.77* | An-LBs |
| 52 | | 266533 | ATP synthase protein MI25 | 22.4/36.7 | 9.50/4.71 | 94 | 9 | | 46 | | + | An-LBs |
| **Cytoskeleton dynamics** | | | | | | | | | | | | |
| 01 | | 54036487 | Tubulin beta-9 chain | 50.6/24.8 | 4.73/6.43 | 73 | 13 | | 33 | | +2.28** | Ls |
| 19 | | 297819272 | Tubulin beta-2 | 51.3/17.2 | 4.73/4.86 | 92 | 11 | | 21 | | +2.33* | An-MBs |
| **Protein synthesis, assembly and degradation** | | | | | | | | | | | | |
| 03 | | 74273101 | Ribosomal protein S4 | 21.4/25.3 | 10.12/5.08 | 72 | 10 | | 61 | | +2.05* | Ls |
| 58 | | 297795465 | kelch repeat-containing protein | 36.2/35.6 | 5.03/5.37 | 85 | 9 | | 21 | | +3.65** | An-LBs |
| **Signal Transduction** | | | | | | | | | | | | |
| 11 | | 240255605 | ETO1(ethylene overproducer 1) | 109.6/32.1 | 5.96/6.42 | 83 | 19 | | 23 | | +1.68* | SBs |
| 56 | | 6624302 | Small GTP-binding protein | 23.5/24.2 | 8.43/5.47 | 72 | 10 | | 52 | | + | An-LBs |
| **DNA processing** | | | | | | | | | | | | |
| 55 | | 55296320 | Putative DNA-(apurinic or apyrimidinic site) lyase (ARP) | 35.4/25.2 | 8.18/5.72 | 77 | | 7 | | 36 | +1.78** | An-LBs |
| **Amino Acid Metabolism** | | | | | | | | | | | | |
| 20 | | 145362282 | Spermidine synthase 1 | 36.8/39.1 | 5.08/4.71 | 116 | 12 | | 53 | | +2.55* | An-MBs |
| **Lipid Metabolism** | | | | | | | | | | | | |
| 53 | | 13124444 | Phospholipase D alpha 1 | 92.2/96.0 | 5.52/5.83 | 215 | 22 | | 37 | | + | An-LBs |
| **Nucleotide Metabolism** | | | | | | | | | | | | |
| 54 | | 15242717 | ADK2（adenosine kinase 2） | 38.2/26.1 | 5.14/5.99 | 113 | 14 | | 50 | | + | An-LBs |
| **Unknown protein** | | | | | | | | | | | | |
| 12 | | 51535085 | Hypothetical protein | 9.0/37.7 | 8.20/5.65 | 85 | 9 | | 70 | | +1.50* | SBs |
| 21 | | 116779313 | Unknown | 12.4/44.1 | 9.46/5.73 | 76 | 7 | | 46 | | +1.88* | An-MBs |
| 21 | | 116779313 | Unknown | 12.4/44.1 | 9.46/5.73 | 76 | 7 | | 46 | | +1.70* | An-LBs |
| 59 | | 159479650 | Hypothetical protein CHLREDRAFT_151447 | 358.8/30.9 | 4.93/6.33 | 75 | 23 | | 8 | | + | An-LBs |
| 60 | | 19439 | Unnamed protein product | 14.3/36.8 | 5.01/4.80 | 75 | 5 | | 50 | | + | An-LBs |
| 61 | | 303276555 | Predicted protein | 46.1/25.6 | 6.19/5.44 | 80 | 14 | | 36 | | +2.26** | An-LBs |
| **Down-regulated** | | | | | | | | | | | | |
| **Cell rescue, defence and virulence** | | | | | | | | | | | | |
| 13 | | 11135407 | Thioredoxin M-type | 19.5/31.2 | 9.41/5.87 | 80 | 10 | | 51 | | -1.50** | SBs |
| 63 | | [113205339](http://www.matrixscience.com/cgi/protein_view.pl?file=../data/20110314/Fttmlaeae.dat&hit=1) | NB-ARC domain containing protein | 93.3/29.0 | 7.05/5.16 | 80 | 14 | | 24 | | - | An-LBs |
| 64 | | 224071423 | NBS-LRR resistance protein | 116.2/47.0 | 9.26/5.08 | 80 | 24 | | 27 | | - | An-LBs |
| 65 | | 75309952 | Glucose and ribitol dehydrogenase homologue 1 | 31.6/32.7 | 6.11/6.28 | 94 | 10 | | 35 | | - | An-LBs |
| 67 | | 4928472 | Type 2 peroxiredoxin | 17.5/15.8 | 5.37/5.59 | 85 | 10 | | 45 | | -2.27** | An-LBs |
| 78 | | 18265381 | Ascorbate peroxidase | 27.8/27.8 | 5.49/6.11 | 154 | 18 | | 67 | | -1.90* | An-LBs |
| 105 | | 81176557 | Annexin-like protein | 36.3/37.8 | 5.44/5.65 | 313 | 33 | | 84 | | -1.90* | An-LBs |
| **Carbohydrate Metabolism** | | | | | | | | | | | | |
| 16 | | 8885622 | N-Glyceraldehyde-2-phosphotransferase-like | 32.0/32.3 | 5.14/4.98 | 103 | 15 | | 40 | | -1.89* | SBs |
| 24 | | 297803740 | UDP-D-glucose/UDP-D-galactose 4-epimerase 2 | 38.7/38.2 | 6.46/6.69 | 103 | 16 | | 45 | | -1.92** | An-MBs |
| 75 | | 21537260 | Putative 2,3-bisphosphoglycerate-independent phosphoglycerate mutase | 60.8/63.0 | 5.51/5.71 | 106 | 21 | | 36 | | -2.10** | An-LBs |
| 76 | | 21537260 | Putative 2,3-bisphosphoglycerate-independent phosphoglycerate mutase | 60.8/63.1 | 5.51/5.79 | 92 | 19 | | 30 | | -2.30** | An-LBs |
| 77 | | 79470337 | Aminomethyltransferase | 43.7/43.6 | 6.30/5.65 | 80 | 10 | | 29 | | - | An-LBs |
| 79 | | S 297824281 | Aconitase C-terminal domain-containing protein | 27.1/24.7 | 6.84/5.27 | 217 | 19 | | 55 | | -2.40** | An-LBs |
| **Energy Metabolism** | | | | | | | | | | | | |
| 07 | | 49359169 | Photosystem II protein | 37.1/33.8 | 6.78/5.23 | 75 | 13 | | 29 | | -1.94* | Ls |
| 25 | | 125972284 | Ribulose-1,5-bisphophate carboxylase/oxygenase large subunit | 50.3/36.7 | 6.04/6.08 | 72 | 13 | | 28 | | - | An-MBs |
| 84 | | 15226453 | AtPPa3 (Arabidopsis thaliana pyrophosphorylase 3) | 25.1/29.9 | 5.55/5.48 | 79 | 8 | | 38 | | - | An-LBs |
| 85 | | 15226453 | AtPPa3 (Arabidopsis thaliana pyrophosphorylase 3) | 25.1/29.8 | 5.55/5.60 | 87 | 11 | | 42 | | - | An-LBs |
| 86 | | 15229530 | ATGSKB6; copper ion binding / glutamate-ammonia ligase | 38.8/39.8 | 5.72/6.16 | 80 | 15 | | 38 | | -2.58* | An-LBs |
| 87 | | 15229530 | ATGSKB6; copper ion binding / glutamate-ammonia ligase | 38.8/42.1 | 5.72/6.47 | 80 | 15 | | 38 | | -3.71** | An-LBs |
| 88 | | 270054998 | Inorganic pyrophosphatase | 24.8/27.8 | 5.91/5.74 | 100 | 9 | | 37 | | -2.18** | An-LBs |
| 89 | | 15231176 | ATPQ (ATP synthase D chain, mitochondrial) | 19.6/17.4 | 5.09/4.85 | 86 | 11 | | 56 | | -2.44** | An-LBs |
| 90 | | 30693102 | EMB1467 (embryo defective 1467); NADH dehydrogenase | 82.2/94.4 | 6.24/6.16 | 177 | 33 | | 40 | | -2.23* | An-LBs |
| 91 | | 11869927 | acetyl-CoA carboxylase 1 | 252.8/57.0 | 6.07/5.24 | 77 | 27 | | 13 | | -3.79** | An-LBs |
| **Wall remodeling and metabolism** | | | | | | | | | | | | |
| 22 | | 15237362 | Reversibly glycosylated polypeptide | 39.0/39.2 | 5.06/4.94 | 120 | 17 | | 42 | | -1.79* | An-MBs |
| 22 | | 15237362 | Reversibly glycosylated polypeptide | 39.0/39.1 | 5.06/4.93 | 120 | 17 | | 42 | | -6.87** | An-LBs |
| 68 | | 15241704 | UDP-glucose 6-dehydrogenase, putative | 53.6/24.0 | 5.60/5.58 | 97 | 11 | | 31 | | - | An-LBs |
| 69 | | 15241704 | UDP-glucose 6-dehydrogenase, putative | 53.6/20.6 | 5.60/6.03 | 124 | 13 | | 35 | | - | An-LBs |
| 70 | | 15241704 | UDP-glucose 6-dehydrogenase, putative | 53.6/54.8 | 5.60/6.21 | 130 | 21 | | 52 | | -4.34* | An-LBs |
| 71 | | 15242316 | UDP-glucose 6-dehydrogenase, putative | 53.6/54.8 | 5.76/6.52 | 152 | 26 | | 53 | | -6.36** | An-LBs |
| 72 | | 15232865 | RGP1( reversibly glycosylated polypeptide 1) | 41.1/40.9 | 5.61/5.58 | 95 | 12 | | 37 | | - | An-LBs |
| 73 | | 15242351 | Reversibly glycosylated polypeptide 2 | 41.4/40.8 | 5.76/5.74 | 164 | 24 | | 61 | | -3.01** | An-LBs |
| **Cytoskeleton dynamics** | | | | | | | | | | | | |
| 14 | 297819272 | | Tubulin beta-2 | 51.3/53.5 | 4.73/5.00 | 285 | 41 | | 64 | | -2.01* | SBs |
| 14 | 297819272 | | Tubulin beta-2 | 51.3/53.5 | 4.73/5.00 | 285 | 41 | | 64 | | -1.55** | An-MBs |
| 31 | 30580468 | | Dynein-1-alpha heavy chain, flagellar inner arm I1 complex | 525.4/22.2 | 5.32/5.76 | 78 | 37 | | 9 | | - | An-MBs |
| 112 | 297793857 | | Tubulin beta-2 | 51.4/55.0 | 4.70/5.01 | 75 | 11 | | 19 | | - | An-LBs |
| 113 | 18409908 | | Actin 3 | 42.1/20.2 | 5.31/4.97 | 96 | 13 | | 46 | | -2.12* | An-LBs |
| 114 | 166582 | | Actin 1 | 42.0/45.0 | 5.31/5.35 | 205 | 27 | | 62 | | -9.77* | An-LBs |
| 115 | 15231447 | | Actin 12 | 42.0/44.2 | 5.37/5.35 | 193 | 25 | | 62 | | -2.39* | An-LBs |
| 116 | 166582 | | Actin-1 | 42.0/18.9 | 5.31/6.41 | 89 | 12 | | 45 | | -2.22** | An-LBs |
| 117 | 159467825 | | Dynein heavy chain 8 | 364.4/28.6 | 5.75/6.31 | 83 | 32 | | 10 | | - | An-LBs |
| 118 | 159476658 | | Cytoplasmic dynein 1b heavy chain | 483.3/20.6 | 6.13/4.46 | 74 | 43 | | 12 | | - | An-LBs |
| **Protein synthesis, assembly and degradation** | | | | | | | | | | | | |
| 08 | 21537296 | | Ribosomal protein L4 | 30.6/31.0 | 8.92/6.24 | 76 | 12 | | 35 | | -1.80* | Ls |
| 32 | 132270 | | Rubber elongation factor protein | 14.7/38.0 | 5.04/6.14 | 74 | 7 | | 61 | | -2.41** | An-MBs |
| 32 | 132270 | | Rubber elongation factor protein | 14.7/38.0 | 5.04/6.14 | 74 | 7 | | 61 | | - | An-LBs |
| 81 | 77999357 | | Protein disulphide isomerase | 55.9/61.0 | 5.00/5.04 | 267 | 23 | | 52 | | -1.71** | An-LBs |
| 80 | 77999357 | | Protein disulphide isomerase | 55.9/58.4 | 5.00/5.07 | 91 | 16 | | 33 | | - | An-LBs |
| 82 | 297839799 | | Metacaspase 7，cysteine-type endopeptidase | 46.0/54.8 | 4.76/4.87 | 105 | 14 | | 27 | | - | An-LBs |
| 83 | 110736416 | | Putative heat shock protein | 90.8/96.6 | 5.26/5.05 | 88 | 19 | | 24 | | - | An-LBs |
| **Signal Transduction** | | | | | | | | | | | | |
| 102 | 168005127 | | CKI3a AtCKI1/2-like cytokinin receptor | 138.3/33.6 | 8.07/4.41 | 80 | 22 | | 22 | | - | An-LBs |
| 103 | 168005127 | | CKI3a AtCKI1/2-like cytokinin receptor | 138.2/37.1 | 8.07/4.93 | 80 | 22 | | 22 | | - | An-LBs |
| 104 | 18394249 | | Transducin family protein / WD-40 repeat family protein | 36.7/36.9 | 5.72/5.55 | 78 | 11 | | 34 | | - | An-LBs |
| **Cellular transport** | | | | | | | | | | | | |
| 29 | 159476424 | | Mu1-Adaptin | 48.8/36.8 | 7.55/5.09 | 79 | 8 | | 25 | | - | An-MBs |
| 66 | 29839389 | | Ferritin-1, chloroplastic | 28.2/25.5 | 5.49/5.18 | 141 | 15 | | 52 | | -1.75** | An-LBs |
| 106 | 115345735 | | Annexin 2 | 36.2/36.6 | 5.76/6.26 | 228 | 32 | | 64 | | -5.96** | An-LBs |
| 111 | 255080776 | | Kinesin heavy chain | 107.7/63.5 | 6.41/6.05 | 73 | 16 | | 16 | | - | An-LBs |
| **DNA processing** | | | | | | | | | | | | |
| 98 | 159472581 | | DNA polymerase zeta | 80.6/34.8 | 9.65/5.93 | 78 | 13 | | 21 | | - | An-LBs |
| 109 | 62902938 | | Maturase K | 51.4/46.1 | 9.58/4.65 | 77 | 9 | | 20 | | - | An-LBs |
| 110 | 45775570 | | Maturase K | 60.8/24.7 | 9.51/4.85 | 83 | 10 | | 21 | | - | An-LBs |
| **Plant development/differentiation** | | | | | | | | | | | | |
| 15 | 2599092 | | WD-40 repeat protein MSI4 | 51.1/64.3 | 5.72/5.91 | 86 | 11 | | 39 | | -1.95* | SBs |
| 23 | 312231793 | | Phytochrome B-1 | 127.5/38.6 | 5.76/5.99 | 80 | 16 | | 17 | | - | An-MBs |
| 107 | 301751677 | | Knotted 1 | 10.3/23.2 | 5.18/5.85 | 78 | 8 | | 81 | | - | An-LBs |
| **Amino Acid Metabolism** | | | | | | | | | | | | |
| 99 | 15239020 | | YUC4 (YUCCA4); monooxygenase/ oxidoreductase | 45.8/42.6 | 9.44/6.31 | 81 | 9 | | 24 | | -2.58±0.59* | An-LBs |
| **Secondary metabolism** | | | | | | | | | | | | |
| 27 | [62126055](http://www.matrixscience.com/cgi/protein_view.pl?file=../data/20110128/FttcIeunt.dat&hit=1) | | Geranylgeranyl reductase | 51.5/66.2 | 9.01/5.90 | 72 | 10 | | 27 | | -6.22** | An-MBs |
| 92 | 86769414 | | Allene oxide synthase 2 | 57.6/49.2 | 8.83/5.25 | 75 | 13 | | 33 | | - | An-LBs |
| 100 | 91694371 | | Caffeoyl-CoA 3-O-methyltransferase | 29.1/33.5 | 5.21/5.15 | 75 | 10 | | 39 | | - | An-LBs |
| 101 | 98991380 | | Flavanone 3-hydroxylase 2 | 40.5/40.2 | 5.37/5.59 | 99 | 12 | | 36 | | - | An-LBs |
| **Lipid Metabolism** | | | | | | | | | | | | |
| 26 | 112490556 | | Chain A, Hmg-Coa Synthase From Brassica Juncea In The Apo-Form | 50.6/46.6 | 6.20/6.22 | 184 | 28 | | 57 | | -1.73* | An-MBs |
| 28 | 294845743 | | Chalcone synthase 2 protein | 43.2/42.6 | 6.00/6.16 | 164 | 24 | | 61 | | -2.02* | An-MBs |
| 93 | 14422255 | | Enoyl-(acyl-carrier protein) reductase | 40.9/37.8 | 9.30/6.48 | 161 | 17 | | 38 | | -2.18* | An-LBs |
| **Nucleotide Metabolism** | | | | | | | | | | | | |
| 94 | 15242717 | | ADK2 (adenosine kinase2) | 38.2/29.0 | 5.14/4.44 | 86 | 13 | | 55 | | - | An-LBs |
| 95 | 30690246 | | PYR6; cytidylate kinase/ uridylate kinase | 22.6/23.5 | 5.79/6.09 | 109 | 11 | | 58 | | -10.58** | An-LBs |
| 96 | 15232763 | | ADK1 (adenosine kinase 1)) | 38.3/42.9 | 5.29/5.38 | 88 | 11 | | 30 | | -1.66** | An-LBs |
| 97 | 15242717 | | ADK2 (adenosine kinase2) | 38.2/43.1 | 5.14/5.18 | 107 | 15 | | 52 | | -1.72* | An-LBs |
| **Unclassified proteins** | | | | | | | | | | | | |
| 17 | 77554545 | | Retrotransposon protein, putative, Ty3-gypsy subclass | 153.2/28.9 | 8.82/4.70 | 101 | 24 | | 17 | | -1.73** | SBs |
| 33 | 30688506 | | Methyltransferase-related | 39.8/39.3 | 6.23/5.31 | 99 | 10 | | 32 | | -2.29** | An-MBs |
| 35 | 30688506 | | Methyltransferase-related | 39.8/56.8 | 6.23/5.74 | 99 | 10 | | 32 | | -2.47* | An-MBs |
| 34 | 30688506 | | Methyltransferase-related | 39.8/38.7 | 6.23/5.48 | 76 | 10 | | 23 | | -2.67* | An-MBs |
| 108 | 42409328 | | Nucleoporin-like protein | 158.0/42.8 | 5.30/4.93 | 88 | 18 | | 17 | | - | An-LBs |
| 120 | 297832912 | | Dimethylmenaquinone methyltransferase family protein | 18.1/17.8 | 5.68/5.30 | 101 | 9 | | 34 | | - | An-LBs |
| **Unknown protein** | | | | | | | | | | | | |
| 09 | 297795029 | | Predicted protein | 47.0/19.3 | 4.92/5.14 | 74 | 8 | | 15 | | -1.50±0.83* | Ls |
| 36 | 297792679 | | Hypothetical protein ARALYDRAFT_495379 | 39.8/41.5 | 5.64/5.64 | 89 | 10 | | 31 | | - | An-MBs |
| 37 | 145355325 | | Predicted protein | 94.4/37.7 | 6.54/4.94 | 73 | 18 | | 19 | | -4.47* | An-MBs |
| 38 | 18415850 | | Unknown protein | 38.8/38.7 | 5.68/5.10 | 75 | 11 | | 23 | | -6.42* | An-MBs |
| 39 | 168062532 | | Predicted protein | 37.5/38.3 | 9.67/5.36 | 72 | 9 | | 40 | | -1.62* | An-MBs |
| 40 | 224138342 | | Predicted protein | 73.6/37.0 | 8.04/5.99 | 77 | 15 | | 31 | | -3.83* | An-MBs |
| 41 | 303283614 | | Predicted protein | 160.2/39.1 | 9.03/5.91 | 74 | 20 | | 16 | | -2.24* | An-MBs |
| 121 | 255552951 | | Conserved hypothetical protein | 40.1/21.2 | 5.79/4.63 | 77 | 11 | | 34 | | - | An-LBs |
| 122 | 297740015 | | Unnamed protein product | 14.7/37.1 | 7.68/5.01 | 78 | 11 | | 31 | | - | An-LBs |
| 123 | 225447009 | | Hypothetical protein | 108.5/28.0 | 6.91/5.06 | 73 | 12 | | 16 | | - | An-LBs |
| 124 | 168061841 | | Predicted protein | 238.8/33.8 | 8.33/5.20 | 80 | 26 | | 17 | | - | An-LBs |
| 125 | 242055697 | | Hypothetical protein SORBIDRAFT_03g046900 | 95.0/57.3 | 8.53/5.14 | 82 | 17 | | 24 | | - | An-LBs |
| 126 | 21741358 | | OSJNBa0032B23.3 | 134.2/29.1 | 9.01/5.80 | 80 | 24 | | 22 | | - | An-LBs |
| 127 | 168021219 | | Predicted protein | 55.6/24.3 | 9.46/6.09 | 81 | 13 | | 33 | | -2.47** | An-LBs |
| 128 | 115461226 | | Os04g0670500 | 53.9/20.0 | 7.74/6.42 | 80 | 13 | | 33 | | -1.61* | An-LBs |
| 129 | 242036443 | | Hypothetical protein SORBIDRAFT_01g042250 | 72.0/27.1 | 8.90/6.08 | 77 | 9 | | 17 | | - | An-LBs |
| 130 | 297811689 | | Hypothetical protein ARALYDRAFT_488394 | 53.6/16.1 | 5.96/5.83 | 106 | 16 | | 31 | | -13.64* | An-LBs |
| 131 | 115444219 | | Os02g0148100 | 42.9/23.6 | 6.70/6.06 | 74 | 9 | | 25 | | -5.54* | An-LBs |

^a^ Number of mass values matched.

^b^ Sequence coverage.

^c^ Spot abundance is expressed as the ratio of intensities of up-regulated (plus value) or down-regulated (minus value) proteins between treatment and control. Fold changes had *P* values. ‘*’ means *P* < 0.05; ‘**’ means *P* < 0.01. ‘-’ represents protein spots detected only in control plant tissues and not in MES-treated ones, and ‘+’ represents protein spots detected only in MES-treated plant tissues and not in control ones.

^d^ ‘Ls,’ leaves; ‘SBs,’ small buds <1 mm long; ‘An-MBs,’ anthers from medium buds 1–3 mm long (from meiosis to the vacuolated-microspore stage); ‘An-LBs,’ anthers from large buds >3 mm long (from the vacuolated-microspore to the mature-pollen stages).
