# Supplementary material for: Cytological and Comparative Proteomic Analyses on Male Sterility in Brassica napus L. Induced by the Chemical Hybridization Agent Monosulphuron Ester Sodium
Source: PLoS One. 2013 Nov 14;8(11):e80191. doi: 10.1371/journal.pone.0080191 (PMC3828188; doi:10.1371/journal.pone.0080191)
Supplement: Table S3 — Corresponding homologues of the 23 unknown proteins. (DOC) [file pone.0080191.s006.doc]

**Table S3.** Corresponding homologues of the 23 unknown proteins

| Spot no. | Gene index.^a^ | Homologue | | | | |
| --- | --- | --- | --- | --- | --- | --- |
|  |  | NCBI  accession no.^b^ | Protein name | Organism | [Identities](http://www.arabidopsis.org/servlets/TairObject?type=locus&name=AT5G01600) ^c^ | [Positives](http://mips.helmholtz-muenchen.de/cgi-bin/proj/funcatDB/search_advanced.pl?action=2&wert=99) ^d^ |
| 09 | 297795029 | NP_192583 | Ulp1 protease family protein | *Arabidopsis thaliana* | [37%](http://www.arabidopsis.org/servlets/TairObject?type=locus&name=AT3G51770) | 49% |
| 12 | 51535085 | ZP_08516850 | Integrase/recombinase | *Corynebacterium bovis* DSM 20582 | [34%](http://www.arabidopsis.org/servlets/TairObject?type=locus&name=AT3G15360) | 51% |
| 21 | 116779313 | ACG25447 | Cytochrome c | *Zea mays* | 88% | 96% |
| 36 | 297792679 | BAB08415 | Spermidine synthase | *A. thaliana* | [93%](http://www.arabidopsis.org/servlets/TairObject?type=locus&name=AT3G15360) | 94% |
| 37 | 145355325 | NP_001694743 | Histone methyltransferase | *Chlamydomonas reinhardtii* | 46% | 60% |
| 38 | 18415850 | XP_003083128 | RNA-binding protein RBM5 and related proteins, contain G-patch and RRM domains (ISS) | *Ostreococcus tauri* | [33%](http://www.arabidopsis.org/servlets/TairObject?type=locus&name=ATMG00860) | [53%](http://mips.helmholtz-muenchen.de/cgi-bin/proj/funcatDB/search_advanced.pl?action=2&wert=99) |
| 39 | 168062532 | XP_002514152 | Graves disease carrier protein | *Ricinus communis* | [58%](http://www.arabidopsis.org/servlets/TairObject?type=locus&name=AT5G36700) | 72% |
| 40 | 162461501 | ACV92697 | Exocyst subunit EXO70 A1 | *Brassica napus* | [86%](http://www.arabidopsis.org/servlets/TairObject?type=locus&name=AT5G62690) | 75% |
| 41 | 303283614 | XP_002889240 | EMB1135 (embryo defective 1135); DNA binding / protein binding / zinc ion binding | *Arabidopsis lyrata* subsp. *lyrata* | [42%](http://www.arabidopsis.org/servlets/TairObject?type=locus&name=AT4G14880) | 57% |
| 59 | 159479650 | CBH12501 | Kinesin K39, putative | *Trypanosoma brucei gambiense* DAL972 | [27%](http://www.arabidopsis.org/servlets/TairObject?type=locus&name=AT1G67500) | [46%](http://mips.helmholtz-muenchen.de/cgi-bin/proj/funcatDB/search_advanced.pl?action=2&wert=99) |
| 60 | 40974917 | P00872 | Ribulose bisphosphate carboxylase small chain SSU1 | *Lemna gibba* | [98%](http://www.arabidopsis.org/servlets/TairObject?type=locus&name=AT4G08150) | [98%](http://mips.helmholtz-muenchen.de/cgi-bin/proj/funcatDB/search_advanced.pl?action=2&wert=99) |
| 61 | 303276555 | CAB11469 | Arginyl-tRNA synthetase | *R. communis* | [57%](http://www.arabidopsis.org/servlets/TairObject?type=locus&name=AT5G25370) | [76%](http://mips.helmholtz-muenchen.de/cgi-bin/proj/funcatDB/search_advanced.pl?action=2&wert=99) |
| 121 | 255552951 | NP_565238 | Kelch repeat-containing F-box family protein | *A. thaliana* | [54%](http://www.arabidopsis.org/servlets/TairObject?type=locus&name=AT3G62510) | [70%](http://mips.helmholtz-muenchen.de/cgi-bin/proj/funcatDB/search_advanced.pl?action=2&wert=99) |
| 122 | 297740015 | XP_002884765 | Pollen ole e 1 allergen and extensin family protein | *A. lyrata* subsp. *Lyrata* | [51%](http://www.arabidopsis.org/servlets/TairObject?type=locus&name=AT4G35860) | [73%](http://mips.helmholtz-muenchen.de/cgi-bin/proj/funcatDB/search_advanced.pl?action=2&wert=99) |
| 123 | 225447009 | NP_850189 | 3'-5' Exonuclease/ nucleic acid binding | *A. thaliana* | [65%](http://www.arabidopsis.org/servlets/TairObject?type=locus&name=AT2G44160) | 80% |
| 124 | 168061841 | NP_001031872 | Exocyst complex component 5 | *A. thaliana* | [45%](http://www.arabidopsis.org/servlets/TairObject?type=locus&name=AT3G07040) | 61% |
| 125 | 242055697 | NP_199029 | Zinc finger protein, putative / regulator of chromosome condensation (RCC1) family protein | *A. thaliana* | [61%](http://www.arabidopsis.org/servlets/TairObject?type=locus&name=AT1G01950) | [76%](http://mips.helmholtz-muenchen.de/cgi-bin/proj/funcatDB/search_advanced.pl?action=2&wert=99) |
| 126 | 21741358 | AAM74438 | Putative retroelement | *Oryza sativa* Japonica Group | [89%](http://www.arabidopsis.org/servlets/TairObject?type=locus&name=AT5G25880) | [91%](http://mips.helmholtz-muenchen.de/cgi-bin/proj/funcatDB/search_advanced.pl?action=2&wert=99) |
| 127 | 168021219 | XP_002529205 | Dead box ATP-dependent RNA helicase, putative | *R. communis* | [73%](http://www.arabidopsis.org/servlets/TairObject?type=locus&name=AT3G07040) | 87% |
| 128 | 115461226 | AAL60579 | Senescence-associated cysteine protease | *Brassica oleracea* | [55%](http://www.arabidopsis.org/servlets/TairObject?type=locus&name=AT3G07040) | 69% |
| 129 | 242036443 | XP_002522392 | Electron transporter, putative | *R. communis* | [44%](http://www.arabidopsis.org/servlets/TairObject?type=locus&name=AT1G67500) | [61%](http://mips.helmholtz-muenchen.de/cgi-bin/proj/funcatDB/search_advanced.pl?action=2&wert=99) |
| 130 | 297811689 | NP_197053 | UDPglucose 6-dehydrogenase | *A. thaliana* | [99%](http://www.arabidopsis.org/servlets/TairObject?type=locus&name=AT3G07040) | 99% |
| 131 | 115444219 | ACD76440 | Mitogen activated protein kinase 14 | *O. sativa* Indica Group | [100%](http://www.arabidopsis.org/servlets/TairObject?type=locus&name=AT3G07040) | [100%](http://www.arabidopsis.org/servlets/TairObject?type=locus&name=AT3G07040) |

BLASTP (NCBI) was used to search the homologues of the unknown proteins in Table S2. The homologues with the highest homology are shown.

*a* Gene index of the unknown proteins listed in Table S2

*b* Accession number of the homologues.

*c* Extent to which two amino acid sequences are invariant.

*d* Similarities based on the scoring matrix use
